# Ionome Dynamics in Grapevine Leaves

**DOI:** 10.3390/plants15132021

**Published:** 2026-06-30

**Authors:** Jozef Kováčik, Marek Vydra, Lenka Husáková, Martina Piroutková, Sławomir Dresler, Martin Dekan, František Duchoň

**Affiliations:** 1 Department of Biology, Faculty of Education, University of Trnava, Priemyselná 4, 918 43 Trnava, Slovakia; 2 Department of Biology and Ecology, Faculty of Natural Sciences, Matej Bel University in Banská Bystrica, Tajovského 40, 974 01 Banská Bystrica, Slovakia; vydra.marek97@gmail.com; 3 Department of Analytical Chemistry, Faculty of Chemical Technology, University of Pardubice, Studentská 573 HB/D, 532 10 Pardubice, Czech Republic; lenka.husakova@upce.cz (L.H.); martina.piroutkova@student.upce.cz (M.P.); 4 Department of Analytical Chemistry, Medical University of Lublin, 4A Chodzki St., 20-093 Lublin, Poland; slawomir.dresler@umlub.edu.pl; 5 Department of Plant Physiology and Biophysics, Institute of Biological Sciences, Faculty of Biology and Biotechnology, Maria Curie-Skłodowska University, 19 Akademicka St., 20-033 Lublin, Poland; 6 Faculty of Electrical Engineering and Information Technology, Slovak University of Technology in Bratislava, Ilkovičova 2961, 841 04 Bratislava, Slovakiafrantisek.duchon@stuba.sk (F.D.)

**Keywords:** heavy metals, mineral nutrition, ontogenesis, soil pollution, vineyard

## Abstract

Despite extensive studies, global patterns governing the accumulation of essential and non-essential elements in grapevine leaves remain insufficiently understood. Therefore, a comprehensive literature survey of 148 studies (selected according to PRISMA 2020 guidelines) was compared with authentic elemental analyses of young and mature leaves of the white cultivar Rhein Riesling and the red cultivar Cabernet Sauvignon to identify the major drivers of grapevine leaf ionome composition. In unstressed grapevine leaves, macroelements followed the concentration order Ca > K > Mg > P, whereas microelements decreased in the order Fe > Mn > Cu > B > Zn > Mo. Seasonal development was associated with opposite trends between Ca and Mg versus K and P. Geographic origin and berry color were associated mainly with differences in Ca, P, Mn, Cu and Fe concentrations. In our authentic samples, potentially toxic elements occurred at substantially lower concentrations than commonly reported in the literature (mainly Pb, Co, As, Cd, Cs, Al, Sr, Ba, Li and Zr). Mature leaves accumulated higher levels of non-essential elements than young leaves, although bioaccumulation from soil remained generally low. Correlation analyses further demonstrated cultivar-dependent relationships among elements, particularly involving Mg. Multivariate analyses revealed that leaf developmental stage represented the primary factor shaping elemental composition, while cultivar-specific effects constituted a secondary but detectable source of variation. Our results identify phenological stage as a dominant factor controlling ionomic composition and suggest that Mg-, Fe-, Zn-, and Cu-associated processes may contribute to the regulation of the accumulation of toxic elements in grapevine leaves.

## 1. Introduction

Grapevine (*Vitis vinifera* L.) is one of the most important fruit crops in the world and, according to available data, is cultivated on an area of approximately 7.5 million hectares worldwide [1]. Its cultivation, among other things, requires the application of fungicides, often copper-based, which can affect the concentration of elements in the soil and in plants [2], and the accumulation of elements is also influenced by soil conditions and geographical location [3].

Plants require a continuous supply of mineral elements throughout their life cycle, particularly essential nutrients that support metabolic functions and are mainly absorbed from the soil [4,5]. In addition, plants may contain elements that are not necessary for their vitality or are directly harmful, including toxic elements (and especially the so-called heavy metals) such as cadmium, lead, chromium, arsenic, tin, cesium, and others [6].

The effect of metals on plants depends on many factors, including the properties of the element, the duration of exposure, the applied concentration, and the plant species itself [7,8,9]. In addition, there is an interaction between essential and non-essential elements in terms of their impact on metabolism [6]. Cultivar-dependent differences in grapevine elemental composition remain poorly resolved, with conflicting reports regarding the accumulation of undesirable elements in white and red cultivars [3,10].

Although there are several works on grapevine focused on elemental profiling of individual cultivars [11], a comprehensive comparison of grapevine in terms of variety color, geographical origin, and ontogenetic stage is lacking. Plant leaves, as the main organ of photosynthesis, require the uptake of elements from the soil through transpiration and are therefore a suitable indicator of elemental contamination in terms of the accumulation of unnecessary or directly toxic elements. Leaf elemental analysis is commonly used to assess environmental pollution, as patterns of element bioaccumulation can help identify potential contamination sources [12].

The aim of this work is, to our knowledge for the first time, to assess the concentration of 30 selected essential and non-essential elements in control (unstressed) grapevine leaves growing in soil and to assess accumulation of elements in this species in terms of various factors (geographic origin, berry color, chlorotype and ontogenetic/phenological stage). In addition to this separation, we also present detailed statistical indicators allowing us to assess the reliability of the data analyzed. As an original aspect, we provide a detailed comparison of our authentic leaf samples of two common cultivars and their ontogenetic stages, assess the bioaccumulation in relation to the element concentration in the respective soil, and compare our data with data extracted from the literature. The results provide a basis for further research into elemental contamination of grapevines from the perspective of cultivars and time.

## 2. Global Overview of the Concentration of Elements in Grapevine Leaves

**Figure 1:**
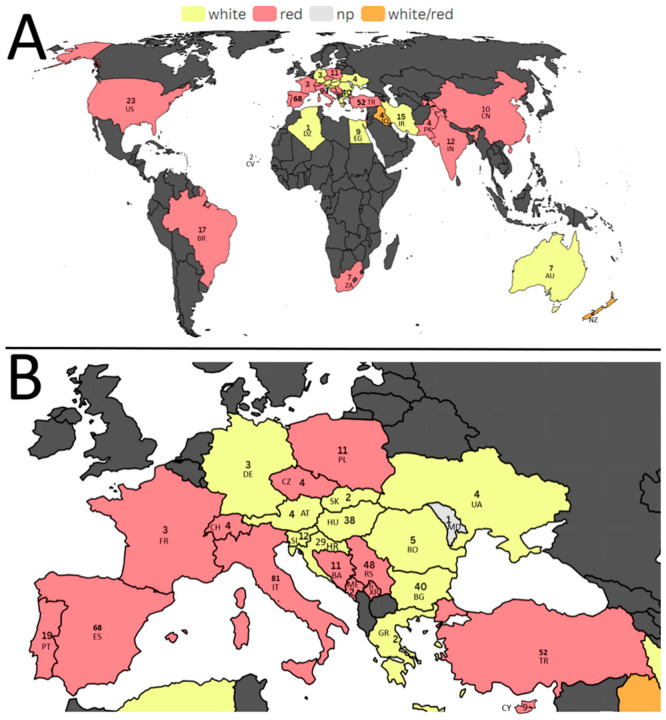
World map (**A**) and a map of most of Europe (**B**) showing the geographical origin of the grapevine cultivars whose leaves were analyzed in papers processed in this review. White = most cultivars analyzed in that country had white berry color; red = most cultivars analyzed in that country had red berry color; np = cultivars analyzed in that country had unspecified berry color; white/red = an equal number of red and white cultivars were analyzed in the given country. The number within each country indicates the total number of unique values we processed in this work (two-letter country codes according to https://countrycode.org/, accessed on 12 March 2026). Data from Tajikistan and Israel are not visible on the map (both countries have only one unique value). In total, 573 unique data across the world were processed (for details, see the Supplementary File “Dataset S1—all quantitatively analyzed studies”).

Analysis of 148 publications and 573 individual quantitative observations revealed that most studies investigating the elemental composition of grapevine leaves were conducted in Europe and Turkey, representing approximately 80% of all records included in the dataset (Figure 1 and supplementary Excel file “Dataset S1—all quantitatively analyzed studies”).

### 2.1. Essential Elements

Based on the overall dataset, we conclude that the quantity of essential macroelements in grapevine leaves is in the descending order Ca > K > Mg > P (Table 1). The concentration of macroelements in leaves in a similar order has generally been observed in individual studies [13,14,15]. Subsequent separation based on geography, cultivar and chlorotype showed significant quantitative differences, especially in the concentration of Ca and P depending on the continent and/or berry color (Figures S2 and S3). In contrast, ontogenetic development exerted a significant effect on leaf macroelement concentrations, characterized by increasing accumulation of Ca and Mg and declining concentrations of K and P during berry ripening (Figures S2 and S3). Such changes in the concentration of macroelements in leaves during individual phenological stages have been observed in individual studies, which indicate a general trend [16,17]. The remobilization of individual elements is often species-specific and influenced by multiple factors, but for example, Ca is characterized by low mobility in plants [18].

The concentration of microelements in leaves followed the descending order Fe > Mn > Cu > B > Zn > Mo based on the overall dataset, although SE values were higher for several elements compared to macroelements (Table 1). Significant differences in Cu values can be explained by the application of conventional fungicides in some studies (as reported, for example, in [3]). In line with these data, the application of copper fungicides to the Sauvignon Blanc cultivar increased Cu concentration in both juice and berry skin, but the largest differences (more than 15-fold) compared to the control group were identified in the leaves [2]. In particular, Mn, Cu, and Fe concentrations differed based on geography or berry color (Figures S4–S6), with red varieties typically containing more of these microelements (Figures S4 and S6). Individual studies did not find such a clear trend for all three elements, but higher Cu [1] and Fe [3] concentrations were observed in red cultivars grown in Italy and the Balkan countries. In contrast, other studies analyzing the concentration of microelements in red and white varieties revealed the opposite or no trend [19,20]. The lack of consistent differences in microelement concentrations indicates that their accumulation is likely governed by a combination of genetic, physiological, and environmental factors. Nevertheless, the elevated concentrations of certain microelements, especially Fe, observed in red cultivars may be partly associated with the enhanced synthesis and metabolism of phenolic compounds in these genotypes (e.g., flavone 3-hydroxylase [21]).

### 2.2. Non-Essential and Toxic Elements

Among the elements analyzed in this work, Na, Al, Sr, Zr, Li, Ba, V, Rb, Ti, Cs, Se, Sb, and Be can be labeled as non-essential (ranked in descending order based on the mean value, Table 1), but they are commonly found in plants in trace amounts. The mean values, especially for Zr, V, and Se due to the wide 95% CI, require further monitoring. Sodium deserves particular attention in continental comparisons, as desertification and increasing aridity can reduce water availability and contribute to soil salinization. Despite the relatively low mean Na concentration observed across the entire dataset (0.724 mg g^−1^ DW; Table 1), European studies consistently reported several-fold lower Na accumulation in grapevine leaves compared with studies from Asia and other continents, suggesting potential regional differences in salinity exposure and/or plant adaptation to local environmental conditions (Figure S3A, Table S4). Conversely, further separations based on cultivar color, chlorotype, and phenological stages did not show clear trends or significant differences between the analyzed groups for some of these elements (Figures S3–S6), although most of them were not analyzed due to a lack of data (Table 1).

Among the toxic elements, the concentrations of Cd, Pb, Cr or As are most often monitored in studies with different plant matrices, and less often Sn or Co accumulation. The overall dataset indicates a high variability in the concentration of most of these elements (min/max values), and in the case of As, a wide range of CI values was also observed (Table 1). The average concentration of these toxic elements was in the order As > Pb > Cr > Co > Cd > Sn (Table 1), and a similar order of concentration of individual elements was generally observed in individual studies [22,23]. Separation based on geography, cultivar color, chlorotype and phenological stage showed differences for Pb and Cr, especially in the latter parameter (Figure S7). Although the obtained average values can be compared with a large amount of individual data from many papers, for comparison we mention similar quantities of elements in leaves of different genera and families of trees in the area of potential pollution, where Cd concentrations up to about 100 ng/g and Pb up to 2.80 µg/g were found [12]. These data indicate similar accumulation of elements resulting from airborne contamination in leaves of woody plants across continents (and bioaccumulation from soil is discussed below in connection with our authentic data).

Although nickel is considered an “ultra-microelement” in plants, it is included in this section due to its potential phytotoxicity, with concentrations above 10 µg/g DW generally regarded as toxic [24]. Analysis of the entire dataset in grapevines showed a mean value of 3.89 µg/g (Table 1), while separation based on different parameters did not reveal clear trends (Figure S7). However, Ni concentration seems to be significantly higher in white cultivars (Figure S7B), which was also found in the white cultivar Italian Riesling [20] and in our authentic data (see discussion below and Table 2).

### 2.3. Correlation Analyses

**Figure 2:**
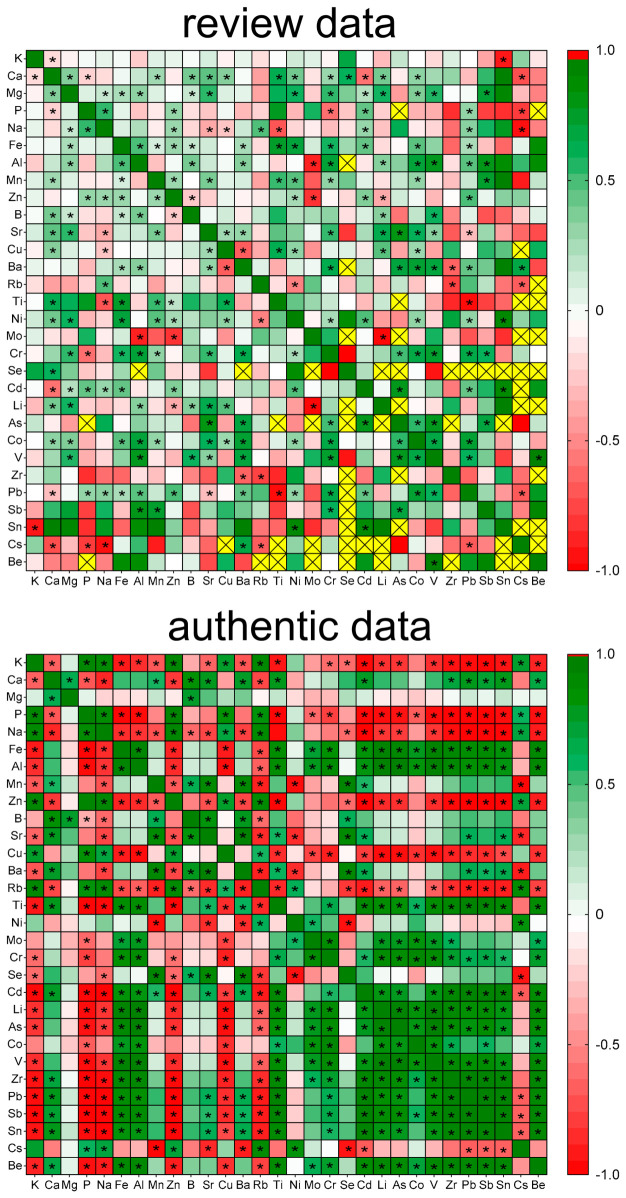
Spearman’s (**top**) and Pearson’s (**bottom**) correlation analyses between the elements using data extracted from the available literature (“review data”) or in our authentic grapevine leaf samples (values from Table 2). Correlations were considered significant at *p* < 0.05 (marked by *). The green and red squares indicate positive or negative correlations, respectively. Combinations without a sufficient data set (less than three) to establish a correlation are crossed out and marked with yellow. For a separate correlation analysis of our authentic white or red grapevine leaf samples, see Supplementary Figure S9.

Correlation analyses of the literature dataset revealed several significant relationships. We identified a negative relationship between K and Ca, as well as between Ca and P and, conversely, a positive correlation between Mg and Ca (Figure 2).

These correlations may partly explain the observed decrease in K and P concentrations accompanied by an increase in Ca and Mg across individual phenological stages (Figures S1 and S2). Consistent with these findings, negative correlations between K and Ca, and positive correlations between Mg and Ca, have been reported, for example, in the white and red cultivars Solaris and Regent [5], and similar relationships were also found in our authentic leaf samples as discussed below (Figure 2). Most non-essential and toxic elements also showed positive or no correlations with each other (Figure 2). We observed no or rather positive correlations between most essential microelements (Fe, Mn, Cu, B, Zn, Mo). The exceptions were the negative correlations between Zn and B/Mo and, conversely, the positive relationship between Zn (an essential element) and some toxic metals (Pb and Cd, Figure 2). The interaction of the microelement boron with other elements and its importance for agricultural production have recently been summarized [25]. However, not all of these relationships were demonstrated in our authentic samples, and given the variability of the literature data (Table 1), we advise the reader to consider the correlations in our authentic data to be more accurate (see Section 4.4).

## 3. Slovak Data: Concentration of Elements in Grapevine Leaves

### 3.1. Essential Elements in Leaves

Among essential macroelements, the concentration of Ca in authentic samples of mature leaves (in contrast to the overall data in Table 1) was only slightly higher compared to K, followed by P and Mg (Table 2), with the quantities being in line with the literature data from Europe and/or the color of the cultivar (Table S4). Our analyses identified a higher concentration of K and P in young leaves of both white and red cultivars (collected at flowering stage, see representative photo of leaves in Figure S8), which may reflect a higher need for osmotic regulation (K) and mitotic activity (P): this is in line with the analysis of the overall dataset, where we identified a higher concentration of these elements in leaves at the flowering stage (Table S4). In agreement, it is known that P deficiency suppresses mitotic activity in leaf meristems by reducing the rate of cell division [26] and that K is typically prevalent in high concentrations in dividing tissues, where it is closely involved in regulating turgor-driven processes [27]. On the contrary, the accumulation of Ca and Mg was higher in mature leaves of the red cultivar (Table 2). A similar trend and therefore a lower concentration of these elements in the flowering phase compared to later stages was also revealed by the analysis of the literature data (Figures S2 and S3). The more intensive mobility of P and K compared to Ca and Mg in grapevine leaves is confirmed not only by the literature but also by our data, especially in the red cultivar (Table 2). Similar changes in the concentration of elements in grapevine leaves during phenological stages were also observed in plants from Spain [16] or Croatia [17].

Microelement concentrations (Fe, Mn, Cu, B, Zn, and Mo) in our authentic samples were broadly consistent with the overall dataset, with the exception of lower mean values for Fe, Mn, and Cu (cf. Table 1 and Table 2). The concentration of elements in mature leaves decreased in the order Fe ≥ Mn ≥ B ≥ Zn > Cu > Mo (Table 2), which is similar to the overall dataset (Table 1). Comparison of mature leaves of cultivars revealed that the concentration of B, Mn and Cu was higher in red grapevine and conversely the concentration of Fe and Mo was higher in white grapevine leaves (Table 2). Higher concentrations of Cu, Mn and also Fe in leaves of red cultivars were also revealed in the overall dataset (Figures S4 and S6). However, individual studies generally did not find higher concentrations of all these elements in red cultivars [28,29], which suggests that their concentration is influenced not only by genetic differences, but probably also by environmental and cultivation factors. Leaf age also affected elemental concentrations, as mature leaves of both cultivars exhibited higher Fe and Mn levels (Table 2). In agreement with these findings, analysis of the complete dataset by phenological stage showed higher Fe concentrations during veraison than during berry set and flowering (Figure S4D).

### 3.2. Non-Essential and Toxic Elements in Leaves

Sodium concentrations in our authentic grapevine leaf samples were approximately 10–20 µg/g (Table 2), compared with the average values of ~120 µg/g reported for Europe and up to ~1200 µg/g in datasets from other continents (Table S4). These discrepancies likely reflect differences in edaphic conditions and may be influenced by regional environmental factors, including climatic conditions. The results obtained in this study (Table 2) agree with published data reporting no significant differences between white and red cultivars (Figure S3C), and indicate that Na concentrations do not vary significantly over the growing season (e.g., [30]) or in the total dataset (Figure S3D). Our authentic data also revealed a lower concentration of common non-essential elements compared to the total dataset, for example in the accumulation of Al, Sr and Ba (Table 1 and Table 2). During ontogenesis, it was shown that most non-essential elements are more accumulated in the tissue of older leaves, and we identified the opposite trend for the elements Rb, Na and Cs (Table 2). Li, Zr, and Cs concentrations in our samples (7.22–103.8 ng/g; Table 2) were markedly lower than the corresponding means in the full dataset (4.93–44.1 µg/g; Table 1). However, the limited dataset did not allow comparison across continents, berry color, or ontogenetic stage. It can be assumed that the low number of available data (and the wide CI, especially for Zr) in combination with possible uncertainties in quantification in the cited papers causes this discrepancy. Further monitoring of some elements is also necessary, as higher accumulation of Li or Cs can be toxic to plants [7,8].

The concentrations of four toxic metals (Pb, Co, As, and Cd) were significantly lower in the authentic samples compared with the mean values derived from the literature. The difference reached up to 100-fold for Cd and was even greater for As (cf. Table 1 and Table 2). In accordance with our data, low Cd concentration (approx. 2–18 ng/g DW) was also found in grapevine leaves from Africa or Europe [20,31]. On the other hand, concentrations of Cr, Ni, and Sn, which can also be classified as potentially toxic elements, were only moderately lower in the authentic samples (up to ~10-fold). For comparison, Co and Ni concentrations in dill leaves from control sites (with a comparable one-year leaf lifespan, as in grapevine), quantified using the same analytical method, were similar to those in our grapevine samples (Table 2), whereas dill exhibited significantly higher concentrations of Cd, Pb, As, Cr, and Sn [6]. In terms of comparing the two cultivars, we found that leaves of the white cultivar contain higher concentrations of four toxic elements at least in the mature stage (Ni, Cr, Co and As), while the concentration of the other three analyzed metals did not differ between the individual cultivars (Pb, Sn and Cd, Table 2). However, previous studies did not report higher toxic metal concentrations in white cultivars [3,10], suggesting cultivar-specific rather than color-dependent accumulation patterns. From an ontogenetic perspective, concentrations of some toxic elements (Cd, Pb, As; Table 2) were higher in mature leaves irrespective of cultivar. Similar patterns have been reported in ontogenetic comparisons of leaves from tree species across different families, suggesting that these elements may be partly derived from atmospheric deposition [12]. The low uptake of these elements from the soil is also reflected by the low BAF values, as mentioned in the next chapter.

### 3.3. Soil Elemental Profile and Bioaccumulation

The concentrations of most analyzed elements in soil did not differ between samples collected in the vicinity of red and white grapevine plants (Table 2), which were grown within the same vineyard located in the peripheral area of Bratislava, the capital city of Slovakia. The absolute amounts of macroelements and microelements in the soil reflect the concentration identified in other recent works with the same methodology in western Slovakia [6,32]. In comparison with other European vineyards where both investigated cultivars were grown, our results indicate lower soil concentrations of Ni, Pb, Cd, Cu, and Zn, comparable levels of Co, Fe, Mn, and K, and higher concentrations of Ca, Na, and As (cf. Table 2 and [3]). However, all our values are well below “the permissible limits of heavy metals in agricultural soil” in the EU or in other countries [33]. Compared with other continents, calcium concentrations in our samples (~7%; Table 2) were substantially higher than those reported, for example, in China (0.52–4.61%; [34]). These differences may partly explain the higher Ca concentrations observed in grapevine leaves grown in Europe compared with those from Asia (Figure S2).

The accumulation potential of plant tissues can be expressed using the bioaccumulation factor (BAF), defined as the tissue-to-soil concentration ratio. BAF values for K, P, and B were close to or above 1, with higher values typically observed in young leaves (Table S6). In accordance with these data, similar BAF values for P or B were identified in the leaves of several cultivars from Italy [35] and Spain [10]. Comparable BAF values were also observed within Slovakia, e.g., in annual plants such as dill [6] or various crops [36]. Many other essential elements (Ca, Mg, Zn, Rb, Cu, Mo) reached values in the range of ca. 0.1–0.6 (Table S6). Similar BAF values for some of these elements have also been reported in several grapevine varieties grown in Spain [10] and Romania [37], including the same cultivars as we examined in this study (BAF values up to 2.19, 0.74, and 1.39 for K, Mg, and Zn, respectively [3]). Among toxic elements, the highest BAF values were observed for Ni (ca. 0.03–0.09) and Cr (0.01–0.02). However, these values were several orders of magnitude lower than those calculated for most essential elements, such as Zn, Co, and Mo (Table S6). BAF values for Pb, Cd, and As were even lower, indicating limited bioaccumulation of these elements in grapevines, consistent with their low absolute concentrations in leaf tissue.

### 3.4. Correlation and PCA Analyses

Correlations between elements in our authentic samples revealed more significant relationships (both positive and negative) compared to literature data on grapevine (Figure 2). Some essential elements, especially K, P, Zn and Cu, showed negative correlations with all toxic elements (Cd, Cr, As, Co, Pb, Sn, Figure 2). Such a clear trend was not identified in other matrices from field conditions, e.g., in dill leaves [6], nor in literature data due to the variability of source data (Figure 2), which suggests that this may be a relationship typical for the analyzed varieties. In addition, individual non-essential and toxic elements generally showed positive correlations with each other, while a similar trend for some of these elements was also observed in the overall dataset. An unexpected finding was the positive correlation between Fe on the one hand and Cr, Cd or Pb on the other hand in all analyzed sets (Figure 2 and Figure S9). Similarly, a positive correlation between Fe and Cd or Pb accumulation in grapevine leaves was also found in a previous study [38], indicating a common soil or anthropogenic origin and/or transport mechanisms. In contrast, Zn or Cu showed predominantly negative correlations with toxic elements in our authentic samples (Figure 2). These data highlight the need for further research into Fe, Zn and Cu metabolism in grapevine in relation to the accumulation of toxic elements. The correlations in our authentic cultivars also revealed variety-specific responses: in particular, the correlations of Mg concentration and partly B, Mo and Co with other elements differed between the white and red cultivars; in the case of Mg, even inverse correlations were found with most elements (Figure S9). These data indicate the need for further research into the selection of suitable cultivars, also with regard to the bioaccumulation of the elements described above.

**Figure 3:**
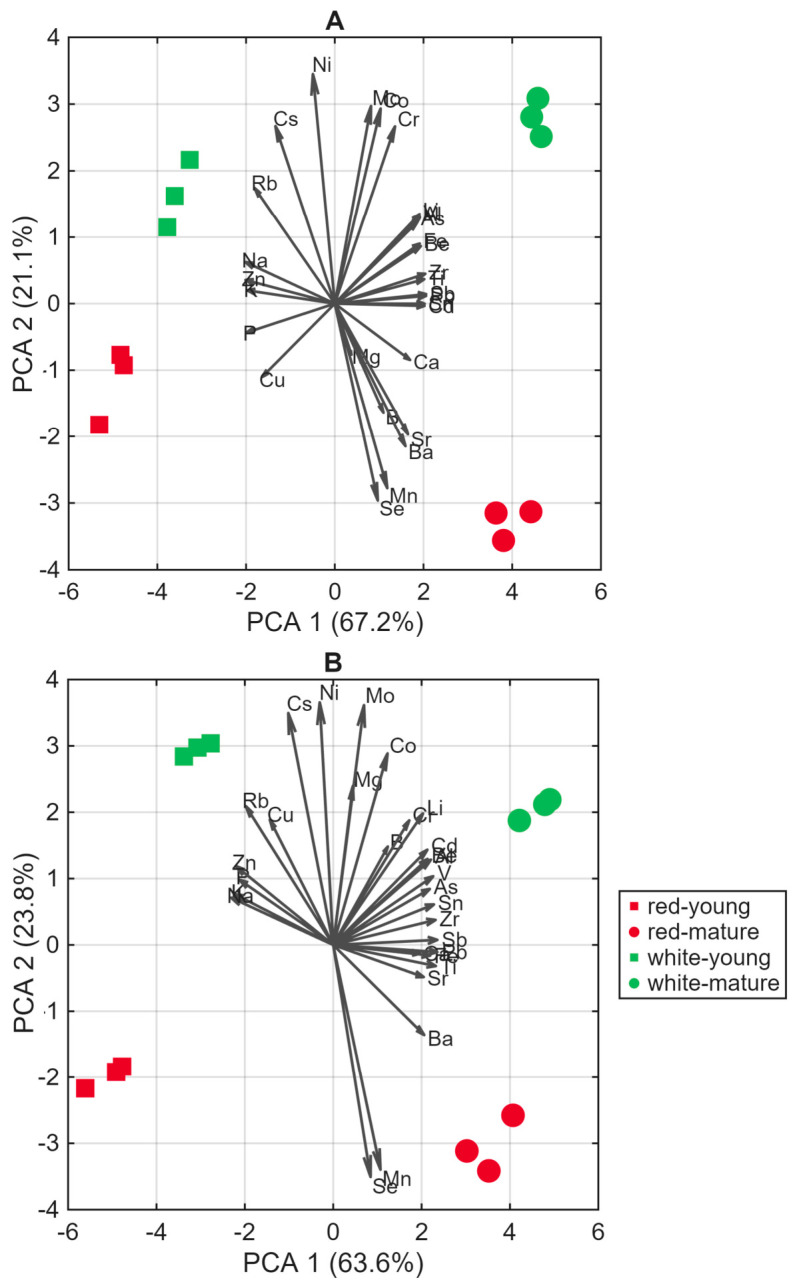
Principal component analysis (PCA) biplots of our authentic grapevine leaf samples according to cultivar (white Rhein Riesling and red Cabernet Sauvignon) and leaf developmental stage (young and mature, see representative photo of leaves in Figure S8 and bioaccumulation factor in Table S6 in Supplementary Materials): (**A**) elemental composition and (**B**) bioaccumulation factors (BAF). The first two principal components explain 88.3% of the total variance in panel (**A**) (PC1 = 67.2%, PC2 = 21.1%) and 87.4% in panel (**B**) (PC1 = 63.6%, PC2 = 23.8%). Points represent individual samples and arrows indicate variable loadings.

Principal component analysis (PCA) of our authentic samples revealed consistent patterns in both the elemental composition and bioaccumulation factor (BAF) datasets of grapevine leaves (Figure 3). In both cases, the first two principal components explained most of the total variance (up to 88%). The dominant source of variability was leaf developmental stage, with a clear separation of young and mature leaves along PC1, where mature leaves were associated with positive PC1 scores and young leaves with negative PC1 values, indicating substantial changes in element accumulation during leaf development and nutrient redistribution within plant tissues [4]. Elements such as Sb, Pb, Zr, Cd, Ti, Fe, and Al showed positive PC1 loadings, whereas K, P, Na, Zn, and Rb were associated with negative PC1 values (Figure S10), suggesting contrasting behavior of trace elements and more mobile nutrient elements in plant metabolism [4]. Cultivar-related differences were expressed mainly along PC2, where elements including Ni, Mo, Co, Cr, and Cs contributed positively to PC2, while Mn, Se, Sr, and Ba showed negative associations (Figure S10A), suggesting cultivar-specific differences in element uptake and accumulation patterns. Comparable trends were observed for the BAF dataset, where elements such as Ti, Pb, Sb, V, Zr, Cd, and Fe contributed positively to PC1, whereas Na, Zn, K, and P showed negative loadings (Figure S10B). Overall, the PCA results indicate that leaf developmental stage represents the primary factor shaping elemental composition and bioaccumulation behavior in grapevine leaves, while cultivar characteristics represent a secondary source of variability.

## 4. Materials and Methods

### 4.1. Literature Search, Screening Process and Coding Scheme

This analysis was performed according to the main recommendations of the PRISMA 2020 guidelines for systematic reviews and meta-analyses [39]. Individual papers were searched for in the Scopus and Web of Science databases because they represent the two main scientific databases of peer-reviewed literature [40].

The search strategy used Boolean logic with the operators “AND” and “OR” to scan titles, abstracts, and keywords of publications. The complete list of keywords and their classification into topics is described in Table S1. The search approach identified 2320 records, for which exclusion and inclusion criteria were subsequently implemented. After selection according to these criteria, 148 full-text articles remained (Figure S1) and the PRISMA 2020 flow diagram was created using the template by Page et al. [41].

From each study, we extracted the country in which the grapevine was grown, the cultivar, the harvest time of the mature leaves, and the reported concentrations of individual elements in the leaves. Only values of individual elements measured in control (unstressed) leaves of *Vitis vinifera* growing in soil were included (if this was stated in the given work). Data presented in graphical form within selected papers were extracted using the WebPlotDigitizer software [42] as this software has been demonstrated to be a reliable tool for extracting data from graphs [43]. The resulting dataset was then categorized according to predefined inclusion criteria for the selected categories (Table S2). In total, we obtained 573 individual quantitative data points on the concentration of selected elements from 148 full-text articles. For a complete list of analyzed papers and the quantity of selected elements, see the supplementary Excel file entitled “Dataset S1—all quantitatively analyzed studies”.

### 4.2. Authentic Slovak Samples

We collected the leaves of a white cultivar (Rhein Riesling) and red cultivar (Cabernet Sauvignon) in May 2025 (flowering stage) in a private vineyard of the Golden Horn winery in Devín, Slovakia (https://www.zlatyroh.sk/). Leaves were collected at two ontogenetic stages as “mature leaves” (fully developed mesophyll) and “young leaves” (about a third of the mature leaf area), approximately the 3rd–4th and 8th–9th leaves from the shoot apex (see representative photo of leaves in Figure S8). The leaves were not rinsed and only leaves without visible damage were collected, which were subsequently dried in an oven at 60 °C to constant weight and ground with an analytical grinder. Leaves from three shoots per plant were pooled into one sample, while leaves from three plants at least 5 m apart were analyzed as three independent samples. Soil was collected near the stem (10–20 cm depth), cleaned of organic and inorganic debris, and dried under the same conditions as leaf samples.

### 4.3. Quantification of Elements in Authentic Samples

Grapevine samples were subjected to microwave-assisted digestion using 5 mL of 16% HNO_3_ and 2 mL of 30% H_2_O_2_, whereas sieved soil samples were treated with aqua regia (7 mL of 37% HCl and 2.5 mL of 65% HNO_3_). Both procedures were conducted at 200 °C for 20 min in a Speedwave XPERT microwave system (Berghof, Eningen, Germany) equipped with optical sensors for contactless real-time monitoring of temperature and pressure in PTFE DAK100 vessels. The resulting grapevine digests were diluted with deionized water to a final volume of 25 mL. Soil extracts were filtered through 0.45 μm nylon syringe filters (Whatman Autovial) and diluted to a final volume of 50 mL. All samples were prepared in triplicate [32]. All measurements were performed using an Agilent 7900 ICP-MS equipped with an octopole-based collision/reaction cell to remove polyatomic interferences. Non-spectral and matrix-related effects were corrected by the online introduction of an internal standard solution containing 200 μg L^−1^ Rh. Instrumental operating conditions are listed in supplementary Table S3. Method detection and quantification limits are reported in Table S3, while method accuracy and precision, assessed using commercially available certified reference materials, are summarized in Table S3. The method provided reliable quantification across different sample matrices over a broad concentration range, from ultra-trace to major element levels. Additional methodological details are the same as previously published [6,44].

### 4.4. Statistical Analysis

GLMs (General Linear Models) were used to determine the influence of the selected factors (world area, berry color, chlorotype and stage of phenological development) on the concentration of elements. Separate GLMs were created for each element (with 50 or more observations), with all factors defined as categorical predictors. The Bonferroni post hoc test (*p* < 0.05) was used to compare multiple categories (groups) of a given factor. All GLMs were performed in the program Statistica^®^ 14.0.0.15. (TIBCO Software Inc. 2020). Data were also tested (at the 0.05 level) for normality using the Shapiro–Wilk test. Because the data did not follow a normal distribution, Spearman’s correlation analyses (at the 0.05 level) were performed to examine relationships between the concentrations of individual elements in *Vitis vinifera* leaves. Graphs and correlation analyses were conducted in GraphPad Prism version 10.6.1 (GraphPad Software, Boston, MA, USA). For the analysis of authentic samples, elemental concentration data were preprocessed prior to multivariate analysis. Each variable was transformed using the Box–Cox transformation to reduce skewness and improve distributional symmetry. The normality of transformed variables was evaluated using the D’Agostino–Pearson omnibus normality test. Following transformation, variables were standardized using z-score normalization (mean-centering and scaling to unit variance) to ensure comparable weighting of all variables in the multivariate analysis. Exploratory multivariate analysis was performed using principal component analysis (PCA). PCA reduces the dimensionality of the dataset by projecting the original variables onto orthogonal principal components that capture the maximum variance in the data. The relationships among samples and variables were visualized using PCA biplots, where sample scores and variable loadings were displayed simultaneously. All statistical calculations and data visualization related to multivariate analysis were performed using MATLAB R2025b (MathWorks, Natick, MA, USA).

## 5. Conclusions and Further Perspectives

Most published grapevine leaf elemental analyses originate from Europe and Turkey, which account for ~80% of the 573 records extracted from 148 studies in this meta-analysis. Across studies, essential macroelements followed the order Ca > K > Mg > P (means: 18.5, 9.36, 3.40, and 2.26 mg/g DW), while microelements followed the order Fe > Mn > Cu > B > Zn > Mo (means: 165, 117, 64.3, 37.4, 34.4, and 0.093 µg/g DW). The meta-analysis revealed clear temporal trends during vegetation, with Ca and Mg increasing during leaf development, while K and P declined, reflecting shifts in nutrient allocation and remobilization. Element concentrations also varied with continent of origin and berry color, particularly for Ca, P, Mn, Cu, and Fe. Among toxic elements, mean concentrations followed the order As > Pb > Cr > Co > Cd > Sn, with phenological stage being the main factor influencing Pb and Cr levels. However, wide confidence intervals for Zr, As, Cd, V, and Se indicate high variability among studies and possible effects of methodological or environmental heterogeneity.

To validate the literature-derived trends, all elements identified in the meta-analysis were quantified in authentic grapevine samples of Rhein Riesling and Cabernet Sauvignon. Concentrations of most essential macro- and microelements agreed well with literature averages, supporting the robustness of the compiled dataset. However, lower levels of Ca, Mg, Fe, Mn, and Cu and higher levels of P and Mo were detected, reflecting cultivar- and site-specific variation. In contrast, non-essential and toxic elements accumulated at substantially lower concentrations than reported in the literature. Levels of Pb, Co, As, Cd, Cs, Al, Sr, Ba, Li, and Zr were generally an order of magnitude lower, with Cd, As, and Pb concentrations being approximately 100-, 300-, and 30-fold lower, respectively. These findings indicate low environmental contamination and limited bioavailability of toxic elements in the studied vineyard despite its proximity to Bratislava, the capital city of Slovakia. They also highlight the potential of grapevine leaves as sensitive bioindicators of environmental quality and trace-element pollution in viticultural regions.

Correlation analyses showed cultivar-dependent element relationships, especially for Mg and to a lesser extent B, Mo, and Co, indicating genotype effects on mineral uptake and homeostasis. Mature leaves also accumulated higher levels of several non-essential elements, suggesting progressive bioaccumulation during development. PCA identified leaf developmental stage as the main driver of the grapevine ionome, with cultivar as a secondary factor.

The results suggest both positive and negative interactions between essential nutrients (notably Mg, Fe, Zn and Cu) and the uptake or retention of toxic elements, with potential implications for vineyard management. Improved nutritional status may help reduce accumulation of undesirable elements in grapevine tissues. Future work should clarify the mechanisms underlying these interactions and assess their relevance under biotic stress and climate change, supporting nutrient strategies that enhance plant health, sustainability, and food safety.

## Figures and Tables

**Table 1:** Descriptive statistics of concentrations of 30 selected elements in grapevine leaves extracted from the available literature (selected according to Table S1 and Figure S1). All data are expressed per g of leaf dry weight. Elements are sorted in descending order based on the average value (mean). N—number of values for a given element, min./max.—range of values found, SE—standard error. For the concentrations of individual elements with respect to world area, berry color, chlorotype and stage of phenological development, see Figures S2–S7 and Table S4 in Supplementary Materials.

	N	Min.	Max.	Mean	SE	Lower 95% CI	Upper 95% CI
Ca (mg/g)	401	0.07	94.8	18.5	0.593	17.3	19.7
K (mg/g)	477	0.1	42.5	9.36	0.263	8.85	9.88
Mg (mg/g)	366	0.01	15.4	3.40	0.098	3.21	3.6
P (mg/g)	352	0.11	8.13	2.26	0.0742	2.12	2.41
Na (mg/g)	156	0.012	14.1	0.724	0.144	0.439	1.01
Fe (mg/g)	388	0.0135	2.83	0.165	0.0113	0.142	0.187
Al (mg/g)	72	0.0189	1.61	0.201	0.0361	0.129	0.273
Mn (mg/g)	331	0.0027	1.03	0.117	0.00699	0.103	0.13
Sr (µg/g)	65	3.46	381	96.2	10.8	74.6	118
Cu (µg/g)	288	0.5	619	64.3	6.41	51.7	76.9
Zr (µg/g)	11	0.052	168	44.1	19.7	0.204	87.9
B (µg/g)	206	6.4	498	37.4	3.15	31.2	43.6
Zn (µg/g)	390	0.02	596	34.4	2.59	29.3	39.5
Li (µg/g)	41	0.01	45	22.7	2.33	18	27.4
Ba (µg/g)	67	1.39	98.2	22.3	2.31	17.6	26.9
As (µg/g)	26	0.001	63	6.44	3.34	−0.431	13.3
V (µg/g)	37	0.001	130	5.70	3.4	−1.33	12.4
Rb (µg/g)	27	1.39	35	5.46	1.18	3.04	7.89
Ti (µg/g)	14	0.12	18.6	5.28	1.63	1.77	8.8
Cs (µg/g)	16	0.029	10.4	4.93	0.787	3.26	6.61
Ni (µg/g)	89	0.04	47.9	3.89	0.773	2.36	5.43
Pb (µg/g)	124	0.0043	40.5	2.88	0.545	1.8	3.96
Cr (µg/g)	64	0.001	6.19	1.77	0.28	1.21	2.34
Co (ng/g)	68	10	3220	685	110	465	904
Cd (ng/g)	133	1	3680	344	58.5	228	459
Se (ng/g)	21	10	2150	180	99.7	−28	388
Sn (ng/g)	6	35.8	180	94.2	24.6	31	157
Mo (ng/g)	26	4.02	500	92.9	25.8	39.8	146
Sb (ng/g)	14	1	51.5	17.1	3.32	9.91	24.2
Be (ng/g)	5	2.36	3.71	2.73	0.249	2.04	3.42

**Table 2:** Concentration of selected elements in leaves of authentic white grapevine (Rhein Riesling) and red grapevine (Cabernet Sauvignon), and in the corresponding soils (pseudo-total concentration). Leaf samples from both ontogenetic stages were collected during the flowering stage (May 2025). All concentrations are expressed on a dry-weight basis (per g of leaf or soil dry weight) and represent mean values of three independent samples. Data are presented in descending order according to the first column on the left. For leaf data, values within a row sharing the same letter(s) are not significantly different according to one-way ANOVA followed by Tukey’s post hoc test (*p* > 0.05). Differences in soil element concentrations were assessed using a *t*-test, with *, **, and *** indicating significance at *p* < 0.05, *p* < 0.01, and *p* < 0.001, respectively. Ratios of individual elements between leaves and soil (bioaccumulation factors) are provided in Table S6 of the Supplementary Materials. A comma within a number indicates tens of thousands.

	White Leaves		Red Leaves		Soil	
	Young	Mature	Young	Mature	White	Red
K (mg/g)	18.7 a	9.10 c	16.1 b	9.81 c	10.8	10.5
Ca (mg/g)	9.27 b	10.5 b	5.38 c	13.4 a	69.3	75.0
P (mg/g)	5.48 a	2.61 c	5.37 a	3.44 b	0.83	1.03
Mg (mg/g)	1.73 a	1.33 b	1.22 b	1.71 a	8.05	10.2
Fe (µg/g)	44.8 c	70.6 a	43.6 c	56.7 b	20,364	18,961
Zn (µg/g)	42.9 a	21.9 b	42.4 a	21.9 b	65.0	70.6
B (µg/g)	36.0 b	34.3 b	30.6 b	43.3 a	32.7	40.1
Mn (µg/g)	32.9 c	47.0 b	49.1 b	78.4 a	549.0	513.8
Rb (µg/g)	30.2 a	9.98 c	23.1 b	2.85 d	71.7	72.5
Na (µg/g)	19.6 a	11.8 b	20.7 a	10.6 b	6332	6176
Al (µg/g)	17.9 c	57.8 a	10.4 d	30.8 b	43,855	47,213
Cu (µg/g)	15.3 a	11.7 b	15.1 a	14.1 a	30.7	36.8
Sr (µg/g)	10.5 c	13.6 b	10.6 bc	29.6 a	154.9	249.9 ***
Ba (µg/g)	2.58 c	3.18 b	2.80 bc	5.04 a	306.51	349.54
Ni (µg/g)	2.08 a	1.96 a	1.39 b	0.74 c	23.9	26.4
Ti (µg/g)	0.83 b	2.16 a	0.42 c	1.92 a	2343	1898 **
Cr (ng/g)	470.5 b	932.9 a	375.0 b	432.2 b	43,852	39,735
Mo (ng/g)	273.3 b	365.9 a	237.6 b	235.4 b	736.6	1550 ***
Cs (ng/g)	103.8 a	57.1 b	60.9 b	18.4 c	3989	5639 ***
Sb (ng/g)	29.6 c	109.6 a	22.0 c	93.9 b	709.8	762.5
V (ng/g)	29.6 c	90.9 a	17.1 d	49.5 b	52,294	50,571
Pb (ng/g)	28.7 b	91.2 a	18.3 b	84.5 a	12,983	12,772
Se (ng/g)	25.1 c	31.9 b	36.4 b	58.5 a	4891	4619
Li (ng/g)	23.4 c	57.5 a	15.5 d	34.3 b	24,035	32,161 *
Co (ng/g)	23.4 b	34.8 a	19.9 b	20.6 b	7612	7980
Sn (ng/g)	10.6 b	17.8 a	8.98 b	17.6 a	2156	2459
As (ng/g)	9.89 c	18.9 a	8.43 c	12.6 b	33,130	32,332
Zr (ng/g)	9.01 c	23.9 a	7.22 c	19.0 b	21,454	22,513
Cd (ng/g)	2.33 b	3.71 a	2.13 b	3.67 a	280.5	371.8 *
Be (ng/g)	0.79 b	1.56 a	0.56 b	1.24 a	1337	1559

## Data Availability

The data processed within meta-analysis are presented in the supplementary Excel file entitled “Dataset S1—all quantitatively analyzed studies”.

## Supplementary Materials

The following supporting information can be downloaded at: https://www.mdpi.com/article/10.3390/plants15132021/s1. Table S1: Terms used in database search (Scopus and Web of Science); Table S2: Criteria for classifying selected data into specific categories of investigated factors; Table S3: Technical details of elemental analyses by ICP-MS; Table S4: Descriptive statistics of the concentration of analyzed elements in specific groups within examined factors (world area, berry color, chlorotype and stage of phenological development); Table S5: Summary results of individual GLMs examining the influence of 4 categorical variables/factors (world area, stage of phenological development, berry color and chlorotype) on the concentration of individual elements; Table S6: The ratio of individual elements in leaves to those in soil (so-called bioaccumulation factor); Figure S1: Methodology for selecting appropriate data from all relevant studies published in the Scopus and Web of Science databases; Figures S2–S7: Separation of selected elements with respect to continent, berry color, chlorotype and stage of phenological development; Figure S8: Representative photos of young and mature leaves of our authentic cultivars; Figure S9: Pearson’s correlation analyses between the elements of our authentic samples: white grapevine (Rhein Riesling) and red grapevine (Cabernet Sauvignon); Figure S10: Heatmaps of loading values for the first two principal components obtained from PCA of grapevine leaves. Refs. [45,46,47,48,49,50,51,52,53,54,55,56,57,58,59,60,61,62,63,64,65,66,67,68,69,70,71,72,73,74,75,76,77,78,79,80,81,82,83,84,85,86,87,88,89,90,91,92,93,94,95,96,97,98,99,100,101,102,103,104,105,106,107,108,109,110,111,112,113,114,115,116,117,118,119,120,121,122,123,124,125,126,127,128,129,130,131,132,133,134,135,136,137,138,139,140,141,142,143,144,145,146,147,148,149,150,151,152,153,154,155,156,157,158,159,160,161,162,163,164,165,166,167,168,169,170,171,172,173,174] are cited in the supplementary Excel file entitled “Dataset S1—all quantitatively analyzed studies” and ref. [175] is cited in the supplementary PDF file.

## References

[1] Cesco S., Tolotti A., Nadalini S., Rizzi S., Valentinuzzi F., Mimmo T., Porfido C., Allegretta I., Giovannini O., Perazzolli M. (2020). *Plasmopara viticola* infection affects mineral elements allocation and distribution in *Vitis vinifera* leaves. Sci. Rep..

[2] Kovačič G.-R., Čuš F., Lesnik M., Pulko B., Valdhuber J., Vršič S. (2016). The impact of copper fungicides on the copper content in organs and wine from a ’Sauvignon Blanc’ grapevine. Mitt. Klosterneubg..

[3] Gulan L., Stajić J.M., Milenković B., Zeremski T., Milić S., Krstić D. (2021). Plant uptake and soil retention of radionuclides and metals in vineyard environments. Environ. Sci. Pollut. Res..

[4] Marschner P. (2012). Marschner’s Mineral Nutrition of Higher Plants.

[5] Lisek J., Popińska W. (2025). Macronutrient status in grapevine leaves and soil in response to fertilizers and biostimulants. Agriculture.

[6] Kováčik J., Husáková L., Vydra M., Piroutková M., Patočka J. (2025). Metallomics of dill: Influence of environmental stress and contamination of commercial samples. J. Environ. Sci..

[7] Shahzad B., Tanveer M., Hassan W., Shah A.N., Anjum S.A., Cheema S.A., Ali I. (2016). Lithium toxicity in plants: Reasons, mechanisms and remediation possibilities—A review. Plant Physiol. Biochem..

[8] Kang D.J., Seo Y.J., Ishii Y. (2019). Distribution of cesium and cationic mineral elements in napiergrass. SN Appl. Sci..

[9] Kováčik J., Vydra M. (2024). The impact of nickel on plant growth and oxidative balance. Physiol. Plant..

[10] Amorós J.A., Pérez-de-los Reyes C., García Navarro F.J., Bravo S., Chacón J.L., Martínez J., Jiménez Ballesta R. (2013). Bioaccumulation of mineral elements in grapevine varieties cultivated in “La Mancha”. J. Plant Nutr. Soil Sci..

[11] Cugnetto A., Santagostini L., Rolle L., Guidoni S., Gerbi V., Novello V. (2014). Tracing the “Terroirs” via the elemental composition of leaves, grapes and derived wines in cv. Nebbiolo (*Vitis vinifera* L.). Sci. Hortic..

[12] Liang J., Fang H.L., Zhang T.L., Wang X.X., Liu Y.D. (2017). Heavy metal in leaves of twelve plant species from seven different areas in Shanghai, China. Urban For. Urban Green..

[13] Likar M., Vogel-Mikuš K., Potisek M., Hančević K., Radić T., Nečemer M., Regvar M. (2015). Importance of soil and vineyard management in the determination of grapevine mineral composition. Sci. Total Environ..

[14] Cancela J.J., Fandiño M., González X.P., Rey B.J., Mirás-Avalos J.M. (2018). Seasonal variation of macro and micronutrients in blades and petioles of *Vitis vinifera* L. cv. Mencía and Sousón. J. Plant Nutr. Soil Sci..

[15] Martínez-Moreno A., Parra M., Intrigliolo D.S., López-Urrea R., Pérez-Álvarez E.P. (2025). Medium-term impacts of saline water deficit irrigation on soil, vine nutrient status, yield and grape composition of *Vitis vinifera* L. cv. Monastrell. Sci. Hortic..

[16] Navarro S., León M., Roca-Pérez L., Boluda R., García-Ferriz L., Pérez-Bermúdez P., Gavidia I. (2008). Characterisation of Bobal and Crujidera grape cultivars, in comparison with Tempranillo and Cabernet Sauvignon: Evolution of leaf macronutrients and berry composition during grape ripening. Food Chem..

[17] Romic M., Zovko M., Romic D., Bakic H. (2012). Improvement of vineyard management of *Vitis vinifera* L. cv. Grk in the Lumbarda vineyard region (Croatia). Commun. Soil Sci. Plant Anal..

[18] Maillard A., Diquélou S., Billard V., Laîné P., Garnica M., Prudent M., Garcia-Mina J.-M., Yvin J.-C., Ourry A. (2015). Leaf mineral nutrient remobilization during leaf senescence and modulation by nutrient deficiency. Front. Plant Sci..

[19] Khan A.S., Ibrahim M., Basra S.M.A., Ali S., Almas M.H., Azam M., Anwar R., Hasan M.U. (2020). Post-bloom productivity and quality of early-season maturing grapes (*Vitis vinifera*). Int. J. Agric. Biol..

[20] Milićević T., Relić D., Škrivanj S., Tešić Ž., Popović A. (2017). Assessment of major and trace element bioavailability in vineyard soil applying different single extraction procedures and pseudo-total digestion. Chemosphere.

[21] Li J.-X., Luo M.-M., Tong C.-L., Zhang D.-J., Zha Q. (2024). Advances in fruit coloring research in grapevine: An overview. Plant Growth Regul..

[22] Milićević T., Urošević M.A., Relić D., Vuković G., Škrivanj S., Popović A. (2018). Bioavailability of potentially toxic elements in soil–grapevine (leaf, skin, pulp and seed) system and environmental and health risk assessment. Sci. Total Environ..

[23] Khaska S., Le Gal La Salle C., Sassine L., Bruguier O., Roig B. (2019). Innovative isotopic method to evaluate bioaccumulation of As and MTEs in *Vitis vinifera*. Sci. Total Environ..

[24] Hassan M.U., Chattha M.U., Khan I., Chattha M.B., Aamer M., Nawaz M., Ali A., Khan M.A.U., Khan T.A. (2019). Nickel toxicity in plants: Reasons, toxic effects, tolerance mechanisms, and remediation possibilities—A review. Environ. Sci. Pollut. Res..

[25] Riaz M., Rafiq M., Nawaz H.H., Miao W. (2025). Bridging molecular insights and agronomic innovations: Cutting-edge strategies for overcoming boron deficiency in sustainable rapeseed cultivation. Plants.

[26] Kavanová M., Lattanzi F.A., Grimoldi A.A., Schnyder H. (2006). Phosphorus deficiency decreases cell division and elongation in grass leaves. Plant Physiol..

[27] Carvalho M.R., Woll A., Niklas K.J. (2016). Spatiotemporal distribution of essential elements through Populus leaf ontogeny. J. Exp. Bot..

[28] Porro D., Bertoldi D., Bottura M., Pedò S. (2022). Five-year period of evaluation of leaf mineral concentrations in resistant varieties in Trentino (northeastern Italy). BIO Web Conf..

[29] Mladenova E., Voyslavov T., Bakardzhiyski I., Karadjova I. (2025). From the soil to the wine—Elements’ migration in monovarietal Bulgarian wines. Molecules.

[30] Calzarano F., Amalfitano C., Seghetti L., Cozzolino V. (2009). Nutritional status of vines affected with esca proper. Phytopathol. Mediterr..

[31] Mahlungulu A., Kambizi L., Akinpelu E.A., Nchu F. (2023). Levels of heavy metals in grapevine soil and leaf samples in response to seasonal change and farming practice in the Cape winelands. Toxics.

[32] Kováčik J., Husáková L., Graziani G., Patočka J., Vydra M., Rouphael Y. (2022). Nickel uptake in hydroponics and elemental profile in relation to cultivation reveal variability in three Hypericum species. Plant Physiol. Biochem..

[33] Srivastava V., Sarkar A., Singh S., Singh P., de Araujo A.S.F., Singh R.P. (2017). Agroecological responses of heavy metal pollution with special emphasis on soil health and plant performances. Front. Environ. Sci..

[34] Wang X., Liu X., Wang W. (2022). National-scale distribution and its influence factors of calcium concentrations in Chinese soils from the China global baselines project. J. Geochem. Explor..

[35] Pepi S., Grisenti P., Sansone L., Chicca M., Vaccaro C. (2018). Chemical elements as fingerprints of geographical origin in cultivars of *Vitis vinifera* L. raised on the same SO_4_ rootstock. Environ. Sci. Pollut. Res..

[36] Kováčik J., Štěrbová D., Babula P., Švec P., Hedbavny J. (2014). Toxicity of naturally-contaminated manganese soil to selected crops. J. Agric. Food Chem..

[37] Bora F.-D., Bunea C.-I., Rusu T., Pop N. (2015). Vertical distribution and analysis of micro-, macroelements and heavy metals in the system soil–grapevine–wine in vineyards from North-West Romania. Chem. Cent. J..

[38] Alagić S.Č., Tošić S.B., Dimitrijević M.D., Nujkić M.M., Papludis A.D., Fogl V.Z. (2018). The content of the potentially toxic elements, iron and manganese, in the grapevine cv. Tamjanika growing near the biggest copper mining/metallurgical complex on the Balkan Peninsula: Phytoremediation, biomonitoring, and some toxicological aspects. Environ. Sci. Pollut. Res..

[39] Moher D., Liberati A., Tetzlaff J., Altman D.G., PRISMA Group (2009). Preferred reporting items for systematic reviews and meta-analyses: The PRISMA statement. PLoS Med..

[40] Pranckutė R. (2021). Web of Science (WoS) and Scopus: The titans of bibliographic information in today’s academic world. Publications.

[41] Page M.J., McKenzie J.E., Bossuyt P.M., Boutron I., Hoffmann T.C., Mulrow C.D., Shamseer L., Tetzlaff J.M., Akl E.A., Brennan S.E. (2021). The PRISMA 2020 statement: An updated guideline for reporting systematic reviews. BMJ.

[42] Rohatgi A. WebPlotDigitizer, Version 5.2; Computer Software, 2015. https://automeris.io/WebPlotDigitizer/.

[43] Aydin O., Yassikaya M.Y. (2022). Validity and reliability analysis of the PlotDigitizer software program for data extraction from single-case graphs. Perspect. Behav. Sci..

[44] Kováčik J., Husáková L., Vlassa M., Piroutková M., Vydra M., Patočka J., Filip M. (2023). Elemental profile identifies metallurgical pollution in epiphytic lichen Xanthoria parietina and (hypo)xanthine correlates with metals. Sci. Total Environ..

[45] Abd El-Khalek A.F., Mazrou Y.S.A., Hatterman-Valenti H.M., Awadeen A.A., El-Mogy S.M.M., El-Kenawy M.A., Belal B.E.A., Mohamed M.A., Hassan I.F., El-Wakeel H.F. (2024). Improvement in physiochemical characteristics of ‘Prime Seedless’ grapes by basal defoliation with foliar-sprayed low-biuret urea and cyanocobalamin under mediterranean climate. Agronomy.

[46] Abou-Zaid E.A.A., Eissa M.A. (2019). Thompson seedless grapevines growth and quality as affected by glutamic acid, vitamin B, and algae. J. Soil Sci. Plant Nutr..

[47] Akin A. (2011). Effects of some growth regulating applications on leaf yield, raw cellulose and nutrient element content of the Müsküle table grape variety. Afr. J. Biotechnol..

[48] Alagić S.Č., Tošić S.B., Dimitrijević M.D., Antonijević M.M., Nujkić M.M. (2015). Assessment of the quality of polluted areas based on the content of heavy metals in different organs of the grapevine (*Vitis vinifera*) cv Tamjanika. Environ. Sci. Pollut. Res..

[49] Aljabary A.M.O., Al-Baytie M.R.S., Ahmed Z.S. (2018). Effect of number eyes leftafter pruning, fertilization with humic acid and spraying with gibberellic acid in some mineral content of vineyards Thompson cv. *Vitis vinifera* L.. Plant Arch..

[50] Al-Saif A.M., Abdel-Hak R.S., Saleh M.M.S., Farouk M.H., Hamed S.R. (2024). Green-nano manganese and its impact on the growth, yield, and fruit properties of flame seedless grapes. Agronomy.

[51] Amorós J.-A., Esbrí J.M., García-Navarro F.-J., Pérez-de-los-Reyes C., Bravo S., Villaseñor B., Higueras P. (2014). Variations in mercury and other trace elements contents in soil and in vine leaves from the Almadén Hg-mining district. J. Soils Sediments.

[52] Angelova V.R., Ivanov A.S., Braikov D.M. (1999). Heavy metals (Pb, Cu, Zn and Cd) in the system soil–Grapevine–Grape. J. Sci. Food Agric..

[53] Arrobas M., Ferreira I.Q., Freitas S., Verdial J., Rodrigues M.Â. (2014). Guidelines for fertilizer use in vineyards based on nutrient content of grapevine parts. Sci. Hortic..

[54] Arrobas M., Thais Nepomuceno Carvalho J., Raimundo S., Poggere G., Rodrigues M.Â. (2022). The safe use of compost derived from municipal solid waste depends on its composition and conditions of application. Soil Use Manag..

[55] Aslanpour M.H.D.B., Baneh H.D., Tehranifar A., Shoor M. (2019). Evaluating the absorption rate of macro and microelements in the leaf of grape Sefid Bidaneh cv. under drought conditions. Int. Trans. J. Eng. Manag..

[56] Banjanin T., Uslu N., Vasic Z.R., Özcan M.M. (2021). Effect of grape varieties on bioactive properties, phenolic composition, and mineral contents of different grape-vine leaves. J. Food Process. Preserv..

[57] Bartkovský M., Semjon B., Regecová I., Baričičová V., Očenáš P., Šuľáková L., Marcinčák S. (2024). The effect of a leaf fertilization method using humic acids on the minerality and chemical composition of Sauvignon Blanc wine from the Slovak wine region. Fermentation.

[58] Bas E.O., Gazioglu Sensoy R.I. (2024). Comparative analysis of salt stress responses in the grapevine (*Vitis vinifera* L.) cultivars: Insights from morphological and physiological assessments. Russ. J. Plant Physiol..

[59] Bavaresco L., Colla R., Fogher C. (2000). Different responses to root infection with endophytic microorganisms of *Vitis vinifera* L. cv. Pinot Blanc grown on calcareous soil. J. Plant Nutr..

[60] Bavaresco L., Fogher C. (1996). Lime-chlorosis occurrence and leaf mineral composition of grapevine treated by root microorganisms. J. Plant Nutr..

[61] Bavaresco L., Giachino E., Pezzutto S. (2003). Grapevine rootstock effects on lime-induced chlorosis, nutrient uptake, and source–sink relationships. J. Plant Nutr..

[62] Bavaresco L., Poni S. (2003). Effect of calcareous soil on photosynthesis rate, mineral nutrition, and source-sink ratio of table grape. J. Plant Nutr..

[63] Bavaresco L., Zamboni M. (1990). Influence of the rootstock and potassium fertilizer on phytoalexin synthesis in Pinot Blanc grown in a calcareous soil. Vitis.

[64] Bayz H.A., Hussein S.A., Ahmed O.I. (2024). Effect of soil mulching and spraying with growth regulator salicylic acid on the characteristics of the mineral content of two cultivars of young grape vines (Halwani and Kamali). IOP Conf. Ser. Earth Environ. Sci..

[65] Benahmed Djilali A., Benseddik A., Boughellout H., Allaf K., Nabiev M. (2021). Biological and functional properties of vine leaves. N. Afr. J. Food Nutr. Res..

[66] Beni C., Rossi G. (2009). Conventional and organic farming: Estimation of some effects on soil, copper accumulation and wine in a central Italy vineyard. Agrochimica.

[67] Benito A., Romero I., Domínguez N., García-Escudero E., Martín I. (2013). Leaf blade and petiole analysis for nutrient diagnosis in *Vitis vinifera* L. cv. Garnacha Tinta: Leaf nutritional diagnosis for Garnacha Tinta vines. Aust. J. Grape Wine Res..

[68] Bertoldi D., Villegas T.R., Larcher R., Santato A., Nicolini G. (2013). Arsenic present in the soil-vine-wine chain in vineyards situated in an old mining area in Trentino, Italy. Environ. Toxicol. Chem..

[69] Brataševec K., Sivilotti P., Vodopivec B.M. (2013). Soil and foliar fertilization affects mineral contents in *Vitis vinifera* L. cv. ‘Rebula’ leaves. J. Soil Sci. Plant Nutr..

[70] Bravo S., Amorós J.A., Pérez-de-los-Reyes C., García F.J., Moreno M.M., Sánchez-Ormeño M., Higueras P. (2017). Influence of the soil pH in the uptake and bioaccumulation of heavy metals (Fe, Zn, Cu, Pb and Mn) and other elements (Ca, K, Al, Sr and Ba) in vine leaves, Castilla-La Mancha (Spain). J. Geochem. Explor..

[71] Brunetto G., Marques A.C.R., Trentin E., Sete P.B., Soares C.R.F.S., Ferreira P.A.A., De Melo G.W.B., Zalamena J., Da Silva L.O.S., Marchezan C. (2023). Arbuscular mycorrhizal fungi inoculation as strategy to mitigate copper toxicity in young field-grown vines. Ciência E Técnica Vitivinícola.

[72] Buesa I., Pérez-Pérez J.G., Visconti F., Strah R., Intrigliolo D.S., Bonet L., Gruden K., Pompe-Novak M., De Paz J.M. (2022). Physiological and transcriptional responses to saline irrigation of young ‘tempranillo’ vines grafted onto different rootstocks. Front. Plant Sci..

[73] Cataldo E., Fucile M., Mattii G.B. (2022). Composting from organic municipal solid waste: A sustainable tool for the environment and to improve grape quality. J. Agric. Sci..

[74] Daccak D., Lidon F.C., Pessoa C.C., Luís I.C., Coelho A.R.F., Marques A.C., Ramalho J.C., Silva M.J., Rodrigues A.P., Guerra M. (2022). Enrichment of grapes with zinc-efficiency of foliar fertilization with ZnSO_4_ and ZnO and implications on winemaking. Plants.

[75] Degaris K.A., Walker R.R., Loveys B.R., Tyerman S.D. (2016). Comparative effects of deficit and partial root-zone drying irrigation techniques using moderately saline water on ion partitioning in Shiraz and Grenache grapevines: Deficit irrigation with saline water. Aust. J. Grape Wine Res..

[76] Dehelean A., Magdas D.A., Cristea G. (2013). Investigation of Trace Metals Content and Carbon Isotopic Composition on the Soil Leaf-Fruit Chain from Some Transylvanian Areas. Anal. Lett..

[77] Demirer T., Müftüoglu N.M., Dardeniz A., Örs T. (2007). Determination of the nutrition standard of soil and leaf analysis of Bozcaada Çavusu grape variety grown in Çanakkale, Turkey. Asian J. Chem..

[78] Di Marco S., Mazzullo A., Cesari A., Osti F. (2001). How Iron Could Be Involved in Esca Fungi Development. Phytopathol. Mediterr..

[79] Dinis L.-T., Correia C.M., Ferreira H.F., Gonçalves B., Gonçalves I., Coutinho J.F., Ferreira M.I., Malheiro A.C., Moutinho-Pereira J. (2014). Physiological and biochemical responses of Semillon and Muscat Blanc à Petits Grains winegrapes grown under Mediterranean climate. Sci. Hortic..

[80] Do Nascimento C.W.A., Da Silva F.B.V., Lima L.H.V., Silva J.R., De Lima Veloso V., Da Silva F.L., De Freitas S.T., Dos Santos L.F., Dos Santos M.A. (2023). Silicon application to soil increases the yield and quality of table grapes (*Vitis vinifera* L.) grown in a semiarid climate of Brazil. Silicon.

[81] Fallahi E., Shafii B., Stark J.C., Fallahi B., Hafez S.L. (2005). Influence of wine grape cultivars on growth and leaf blade and petiole mineral nutrients. HortTechnology.

[82] Farouk S., Belal B.E.A., EL-Sharkawy H.H.A. (2017). The role of some elicitors on the management of Roumy Ahmar grapevines downy mildew disease and it’s related to inducing growth and yield characters. Sci. Hortic..

[83] Gąstol M., Domagała-Świątkiewicz I. (2016). Trace element partitioning in ‘Sibera’ grapevines as affected by nitrogen fertilisation. S. Afr. J. Enol. Vitic..

[84] Gil-Pérez B., Zarco-Tejada P.J., Correa-Guimaraes A., Relea-Gangas E., Navas-Gracia L.M., Hernández-Navarro S., Sanz-Requena J.F., Berjón A., Martín-Gil J. (2010). Remote sensing detection of nutrient uptake in vineyards using narrow-band hyperspectral imagery. Vitis.

[85] Goodarzi K., Hosseini Farahi M. (2014). Evaluating the ability of sulfur and animal manure to relieve Fe, Mn, Zn, Cu and B deficiency in “Seah” table grapes in Cisakht region of Iran. Acta Hortic..

[86] Güneş A., Köse C., Turan M. (2015). Yield and mineral composition of grapevine (*Vitis vinifera* L. cv. Karaerik) as affected by boron management. Turk. J. Agric. For..

[87] Hirzel D.R., Steenwerth K., Parikh S.J., Oberholster A. (2017). Impact of winery wastewater irrigation on soil, grape and wine composition. Agric. Water Manag..

[88] Howell C.L., Myburgh P.A., Lategan E.L., Schoeman C., Hoffman J.E. (2016). Effect of irrigation using diluted winery wastewater on *Vitis vinifera* L. cv. Cabernet Sauvignon in a sandy alluvial soil in the breede river valley–vegetative growth, yield and wine quality. S. Afr. J. Enol. Vitic..

[89] Hummes A.P., Bortoluzzi E.C., Tonini V., da Silva L.P., Petry C. (2019). Transfer of copper and zinc from soil to grapevine-derived products in young and centenarian vineyards. Water Air Soil Pollut..

[90] Chopin E.I.B., Marin B., Mkoungafoko R., Rigaux A., Hopgood M.J., Delannoy E., Cancès B., Laurain M. (2008). Factors affecting distribution and mobility of trace elements (Cu, Pb, Zn) in a perennial grapevine (*Vitis vinifera* L.) in the Champagne region of France. Environ. Pollut..

[91] Iacono F., Porro A.D., Scienza A., Stringari G. (1995). Differential effects of canopy manipulation and shading of *Vitis vinifera* L. cv. Cabernet Sauvignon: Plant nutritional status. J. Plant Nutr..

[92] Irani H., ValizadehKaji B., Naeini M.R. (2021). Biostimulant-induced drought tolerance in grapevine is associated with physiological and biochemical changes. Chem. Biol. Technol. Agric..

[93] Kamiloğlu Ö. (2022). Impact of rootstocks on fruit quality, mineral nutrition and leaf physiology of ‘Red Globe’ in the East Mediterranean region. Appl. Ecol. Environ. Res..

[94] Karažija T., Štimac M., Petek M., Šatvar M., Lazarević B. (2021). Long-term effects of organic fertilizers on microelements status in grapevine leaf on calcareous soil. Sci. Pap. Ser. B Hortic..

[95] Karimi R. (2017). Potassium-induced freezing tolerance is associated with endogenous abscisic acid, polyamines and soluble sugars changes in grapevine. Sci. Hortic..

[96] Karimi R., Ghabooli M., Rahimi J., Amerian M. (2020). Effects of foliar selenium application on some physiological and phytochemical parameters of *Vitis vinifera* L. cv. Sultana under salt stress. J. Plant Nutr..

[97] Kaya G., Yaman M. (2012). Determination of trace metals in plant leaves as biomonitor of pollution extent by a sensitive Stat-AAS method. Instrum. Sci. Technol..

[98] Kocsis L., Lehoczky É. (2000). Applications in sustainable production: The effect of the graperootstock-scion interaction on the potassium and calcium content of the leaves in connection with yield production. Commun. Soil Sci. Plant Anal..

[99] Kocsis L., Lehoczky É. (2002). The significance of yield production and sugar content of the grapejuice with macronutrients in grape rootstock–scion combinations on dry climatic condition. Commun. Soil Sci. Plant Anal..

[100] Kőrösi L., Bognár B., Czégény G., Lauciello S. (2022). Phase-selective synthesis of anatase and rutile TiO_2_ nanocrystals and their impacts on grapevine leaves: Accumulation of mineral nutrients and triggering the plant defense. Nanomaterials.

[101] Lai H.-Y., Juang K.-W., Chen B.-C. (2010). Copper concentrations in grapevines and vineyard soils in central Taiwan. Soil Sci. Plant Nutr..

[102] Likar M., Stres B., Rusjan D., Vogel-Mikuš K., Regvar M. (2022). Grapevine leaf ionome is shaped by soil factors and plant age. Plant Soil Environ..

[103] Lyu H., Grafton M., Ramilan T., Irwin M., Sandoval E. (2023). Assessing the leaf blade nutrient status of Pinot Noir using hyperspectral reflectance and machine learning models. Remote Sens..

[104] Ma J., Zhang M., Liu Z., Chen H., Li Y.C., Sun Y., Ma Q., Zhao C. (2019). Effects of foliar application of the mixture of copper and chelated iron on the yield, quality, photosynthesis, and microelement concentration of table grape (*Vitis vinifera* L.). Sci. Hortic..

[105] Maia M., Cavaco A.R., Laureano G., Cunha J., Eiras-Dias J., Matos A.R., Duarte B., Figueiredo A. (2021). More than just wine: The nutritional benefits of grapevine leaves. Foods.

[106] Marques R., Prudêncio M.I., Abreu M.M., Russo D., Marques J.G., Rocha F. (2019). Chemical characterization of vines grown in incipient volcanic soils of Fogo Island (Cape Verde). Environ. Monit. Assess..

[107] Mercurio M., Grilli E., Odierna P., Morra V., Prohaska T., Coppola E., Grifa C., Buondonno A., Langella A. (2014). A ‘Geo-Pedo-Fingerprint’ (GPF) as a tracer to detect univocal parent material-to-wine production chain in high quality vineyard districts, Campi Flegrei (Southern Italy). Geoderma.

[108] Mostashari M., Khosravinejad A., Golmohammadi M. (2018). Comparative study of DOP and CND methods for leaf nutritional diagnosis of *Vitis vinifera* in Iran. Commun. Soil Sci. Plant Anal..

[109] Naegele R.P., Londo J.P., Zou C., Cousins P. (2021). Identification of SNPs associated with magnesium and sodium uptake and the effect of their accumulation on micro and macro nutrient levels in *Vitis vinifera*. PeerJ.

[110] Nagy P.T., Pintér T. (2015). Effects of foliar biofertilizer sprays on nutrient uptake, yield, and quality parameters of Blaufrankish (*Vitis vinifera* L.) grapes. Commun. Soil Sci. Plant Anal..

[111] Nakajima H., Behboudian M.H., Greven M., Zegbe-Domínguez J.A. (2004). Mineral contents of grape, olive, apple, and tomato under reduced irrigation. J. Plant Nutr. Soil Sci..

[112] Niemiec M., Niemiec M., Chowaniak M., Komorowska M., Zuzek D., Saidali Mamurovich G., Kodirov Gafurovich K., Usmanov N., Kamilova D., Rahmonova J. (2020). Evaluation of the chemical composition of soil as well as vine leaves and berries from the selected commercial farms in the republic of Tajikistan. J. Elem..

[113] Nikolaou N., Karagiannidis N., Koundouras S., Fysarakis I. (2002). Effects of different P-sources in soil on increasing growth and mineral uptake of mycorrhizal *Vitis vinifera* L. (cv Victoria) vines. OENO One.

[114] Nitin P.S., Patel V.B., Singh S.K., Verma M.K., Mishra G.P., Anil D., Puneeth P.V. (2025). Stionic influence of grape cultivar Syrah (*Vitis vinifera* L.) on inter-specific hybrid rootstocks. S. Afr. J. Enol. Vitic..

[115] Nogales A., Santos E.S., Abreu M.M., Arán D., Victorino G., Pereira H.S., Lopes C.M., Viegas W. (2019). Mycorrhizal inoculation differentially affects grapevine’s performance in copper contaminated and non-contaminated soils. Front. Plant Sci..

[116] Oliveira V.D.S., Lima A.M.N., Salviano A.M., Bassoi L.H., Pereira G.E. (2015). Heavy metals and micronutrients in the soil and grapevine under different irrigation strategies. Rev. Bras. Ciência Solo.

[117] Ortiz-Villajos J.A.A., Navarro F.J.G., Martín-Consuegra S.B., Ballesta R.J., Moreno R.G. (2012). Geochemical influence of soil on leaf and grape (*Vitis vinifera* L. ’Cencibel’) composition in La Mancha region (Spain). Vitis.

[118] Özdemir G., Tangolar S. (2007). Effect of iron applications on Fe, Zn, Cu and Mn compositions of grapevine leaves. Asian J. Chem..

[119] Pachnowska K., Ochmian I. (2018). Influence of rootstock on elemental composition in leaves and grapes of vine cultivar ‘Regent’ grown in North-Western Poland. J. Appl. Bot. Food Qual..

[120] Pantelić M.M., Zagorac D.Č.D., Ćirić I.Ž., Pergal M.V., Relić D.J., Todić S.R., Natić M.M. (2017). Phenolic profiles, antioxidant activity and minerals in leaves of different grapevine varieties grown in Serbia. J. Food Compos. Anal..

[121] Pepi S., Chicca M., Piroddi G., Tassinari R., Vaccaro C. (2019). Geographical origin of *Vitis vinifera* cv. Cannonau established by the index of bioaccumulation and translocation coefficients. Environ. Monit. Assess..

[122] Pérez-De-Los-Reyes C., Ortíz-Villajos J.A.A., Navarro F.J.G., Martín-Consuegra S.B., Ballesta R.J. (2013). Grapevine leaf uptake of mineral elements influenced by sugar foam amendment of an acidic soil. Vitis J. Grapevine Res..

[123] Peršurić Palčić A., Jeromel A., Pecina M., Palčić I., Gluhić D., Petek M., Herak Ćustić M. (2022). Decreased leaf potassium content affects the chemical composition of must for sparkling wine production. Horticulturae.

[124] Peuke A.D. (2009). Nutrient composition of leaves and fruit juice of grapevine as affected by soil and nitrogen fertilization. J. Plant Nutr. Soil Sci..

[125] Pinamonti F. (1998). Compost mulch effects on soil fertility, nutritional status and performance of grapevine. Nutr. Cycl. Agroecosyst..

[126] Porro D., Dallaserra M., Zatelli A., Ceschini A. (2001). The interaction between nitrogen and shade on grapevine: The effects on nutritional status, leaf age and leaf gas exchanges. Acta Hortic..

[127] Porro D., Ramponi M., Tomasi T., Rolle L., Poni S. (2010). Nutritional implications of water stress in grapevine and modifications of mechanical properties of berries. Acta Hortic..

[128] Prodanova-Marinova N., Staneva I., Tsvetanov E. (2023). Competitive relations between young vines and weed species for mineral nutrients uptake in the nursery. Bulg. J. Agric. Sci..

[129] Quartacci M.F., Ranieri A., Sgherri C. (2015). Antioxidative defence mechanisms in two grapevine (*Vitis vinifera* L.) cultivars grown under boron excess in the irrigation water. Vitis.

[130] Rasouli M., Bayanati M., Tavakoli F. (2024). Improving quantitative and qualitative traits of grapes cv. ‘Fakhri’ of Iran with foliar application of potassium silicate and humic acid. Russ. J. Plant Physiol..

[131] Reeve J.R., Carpenter-Boggs L., Reganold J.P., York A.L., McGourty G., McCloskey L.P. (2005). Soil and winegrape quality in biodynamically and organically managed vineyards. Am. J. Enol. Vitic..

[132] Reta K., Lazarovitch N., Fait A. (2025). Metabolic and physiological analysis reveals distinct salinity tipping point in *Vitis vinifera* cv. Syrah to enter a stress response mode. Plant Stress.

[133] Richardson J.B., Chase J.K. (2021). Transfer of macronutrients, micronutrients, and toxic elements from soil to grapes to white wines in uncontaminated vineyards. Int. J. Environ. Res. Public Health.

[134] Romero I., Benito A., Dominguez N., Garcia-Escudero E., Martin I. (2014). Leaf blade and petiole nutritional diagnosis for *Vitis vinifera* L. cv. Tempranillo by deviation from optimum percentage method. Span. J. Agric. Res..

[135] Romero I., García-Escudero E., Martín I. (2010). Effects of leaf position on blade and petiole mineral nutrient concentration of Tempranillo grapevine (*Vitis vinifera* L.). Am. J. Enol. Vitic..

[136] Romero P., Botía P., Gil-Muñoz R., Del Amor F.M., Navarro J.M. (2023). Evaluation of the effect of water stress on clonal variations of Cv. Monastrell (*Vitis vinifera* L.) in south-eastern Spain: Physiology, nutrition, yield, berry, and wine-quality responses. Agronomy.

[137] Rozane D.E., Vahl De Paula B., Wellington Bastos De Melo G., Haitzmann Dos Santos E.M., Trentin E., Marchezan C., Stefanello Da Silva L.O., Tassinari A., Dotto L., Nunes De Oliveira F. (2020). Compositional nutrient diagnosis (CND) applied to grapevines grown in subtropical climate region. Horticulturae.

[138] Sabir A. (2016). Vegetative and reproductive growth responses of grapevine cv. “Italia” (*Vitis vinifera* L.) grafted on different rootstocks to contrasting soil water status. J. Agric. Sci. Technol..

[139] Sabir A., Yazar K., Sabir F., Kara Z., Yazici M.A., Goksu N. (2014). Vine growth, yield, berry quality attributes and leaf nutrient content of grapevines as influenced by seaweed extract (*Ascophyllum nodosum*) and nanosize fertilizer pulverizations. Sci. Hortic..

[140] Sala F., Camen D., Herbei M.V., Blidariu C. (2024). Analysis of vine nutrition and productivity based on statistical indicators. Horticulturae.

[141] Salih Z.R., Khudhur N.S., Muhammad M.Q. (2025). Bioaccumulation of different heavy metals and toxicity assessment using different indices in grape plants and soil around power generators in Erbil province. Environ. Monit. Assess..

[142] Sedláček M., Pavloušek P., Lošák T., Zatloukalová A., Filipčík R., Hlušek J., Vítězová M. (2013). The effect of arbuscular mycorrhizal fungi on the content of macro and micro elements in grapevine (*Vitis vinifera* L.) leaves. Acta Univ. Agric. Silvic. Mendel. Brun..

[143] Schreiner R.P., Lee J. (2014). Effects of post-véraison water deficit on ‘Pinot noir’ yield and nutrient status in leaves, clusters, and musts. HortScience.

[144] Sharma J., Upadhyay A.K., Sawant S.D., Sawant I.S. (2009). Studies on shiny spot symptom development on grapevine leaves and its effect on fruitfulness, disease incidence and vine yield. Indian J. Hortic..

[145] Si P., Shao W., Yu H., Xu G., Du G. (2022). Differences in Microbial communities stimulated by malic acid have the potential to improve nutrient absorption and fruit quality of grapes. Front. Microbiol..

[146] Soja G., Wimmer B., Rosner F., Faber F., Dersch G., Von Chamier J., Pardeller G., Ameur D., Keiblinger K., Zehetner F. (2018). Compost and biochar interactions with copper immobilisation in copper-enriched vineyard soils. Appl. Geochem..

[147] Squeri C., Gatti M., Garavani A., Vercesi A., Buzzi M., Croci M., Calegari F., Vincini M., Poni S. (2019). Ground truthing and physiological validation of Vis-NIR spectral indices for early diagnosis of nitrogen deficiency in cv. Barbera (*Vitis vinifera* L.) grapevines. Agronomy.

[148] Stanimirovic B., Vujovic D., Pejin B., Popovic Djordjevic J., Maletic R., Raicevic P., Tesic Z. (2019). A contribution to the elemental profile of the leaf samples of newly developed Cabernet Franc varieties. Nat. Prod. Res..

[149] Domagała-Świątkiewicz I., Gąstoł M. (2013). Effect of nitrogen fertilization on the content of trace elements in cv. Bianca grapevine (*Vitis* sp.). J. Elem..

[150] Taghavi T., Hoseinabadi H., Solgi M., Askari M., Rahemi A. (2020). Influence of vinegar and chelated iron field sprays on mineral nutrients and fruit quality of grapes (cv. ’Thompson Seedless’). Mitteilungen Klosterneubg..

[151] Tan S., Crabtree G.D. (1990). Competition between perennial ryegrass sod and ‘Chardonnay’ wine grapes for mineral nutrients. HortScience.

[152] Tangolar S., Tangolar S., Alkan Torun A., Ada M., Göçmez S. (2020). Influence of supplementation of vineyard soil with organic substances on nutritional status, yield and quality of ‘Black Magic’ grape (*Vitis vinifera* L.) and soil microbiological and biochemical characteristics. OENO One.

[153] Thum A.B., Arruda D.C., Ducati J.R., Pithan P.A., Rolim S.B.A. (2020). The influence of mineral content on spectral features of vine leaves. Int. J. Remote Sens..

[154] Tonev D., Geleva E., Damianova A., Grigorov T., Goutev N., Protohristov H., Stoyanov C., Bashev V., Popov E., Tringovska I. (2016). Radiological and microanalytical studies of fine Melnik wines. C. R. L’Acad. Bulg. Sci..

[155] Topalovic A., Godjevac D., Perovic N., Trifunovic S. (2012). Comparative study of the phenolic composition of seeds from grapes cv Cardinal and Alphonse Lavallee during last month of ripening. Ital. J. Food Sci..

[156] Toselli M., Baldi E., Marcolini G., Malaguti D., Quartieri M., Sorrenti G., Marangoni B. (2009). Response of potted grapevines to increasing soil copper concentration. Aust. J. Grape Wine Res..

[157] Tutus A., Gazioglu Sensoy R.I. (2024). Pruning and fertilization impact on leaf-mineral composition in high-altitude cultivation of grapevines (*Vitis vinifera* L.). Appl. Fruit Sci..

[158] Tzortzakis N., Chrysargyris A. (2024). Olive-mill and grape-mill residue impact the growth, physiology and nutrient status of grapevines young cuttings. Sustain. Chem. Pharm..

[159] Tzortzakis N., Chrysargyris A. (2025). Alternative growing media under the same fertigation scheme affected mineral accumulation and physiological parameters in grapevine cultivars. Horticulturae.

[160] Úbeda X., Francos M., Eguzkiza P., Stefanuto E.B. (2021). Soil and grapevine leaf quality in organic vineyards of different ages in DO Rioja-Alavesa, northern Spain. Span. J. Soil Sci..

[161] Verdenal T., Zufferey V., Dienes-Nagy Á., Bieri S., Bourdin G., Reynard J.-S., Spring J.-L. (2024). Exploring grapevine canopy management: Effects of removing main leaves or lateral shoots before flowering. OENO One.

[162] Victorino G., Santos E.S., Abreu M.M., Viegas W., Nogales A. (2021). Detrimental effects of copper and EDTA co-application on grapevine root growth and nutrient balance. Rhizosphere.

[163] Vystavna Y., Rätsep R., Klymenko N., Drozd O., Pidlisnyuk V., Klymenko M. (2015). Comparison of soil-to-root transfer and translocation coefficients of trace elements in vines of Chardonnay and Muscat white grown in the same vineyard. Sci. Hortic..

[164] Vystavna Y., Rushenko L., Diadin D., Klymenko O., Klymenko M. (2014). Trace metals in wine and vineyard environment in southern Ukraine. Food Chem..

[165] Vystavna Y., Schmidt S.I., Klimenko O.E., Plugatar Y.V., Klimenko N.I., Klimenko N.N. (2020). Species-dependent effect of cover cropping on trace elements and nutrients in vineyard soil and *Vitis*. J. Sci. Food Agric..

[166] Walker R.R., Blackmore D.H., Clingeleffer P.R., Correll R.L. (2004). Rootstock effects on salt tolerance of irrigated field-grown grapevines (*Vitis vinifera* L. cv. Sultana) 2. Ion concentrations in leaves and juice. Aust. J. Grape Wine Res..

[167] Yang Y., Fang X., Chen M., Wang L., Xia J., Wang Z., Fang J., Tran L.-S.P., Shangguan L. (2022). Copper stress in grapevine: Consequences, responses, and a novel mitigation strategy using 5-aminolevulinic acid. Environ. Pollut..

[168] Yildiz H., Cakir O., Cakiroglu K., Karatas N. (2024). A comparative study on the bioactivity and mineral content of different grapevine (*Vitis vinifera* L.) leaves cultivated in Türkiye. Appl. Fruit. Sci..

[169] Yumuşakbaş H., Uğur Y., Maraş Z., Büyüksoylu S., Erdoğan S. (2025). Assessment of heavy metal accumulation and essential nutrients in fruits: Implications for food safety and environmental sustainability. Environ. Monit. Assess..

[170] Zareei E., Zaare-Nahandi F., Oustan S., Hajilou J., Dadpour M. (2022). Insight into the role of magnetic nutrient solution on leaf morphology and biochemical attributes of Rasha grapevine (*Vitis vinifera* L.). Plant Physiol. Biochem..

[171] Zhang P., Dong T., Jin H., Pei D., Pervaiz T., Ren Y., Jia H., Fang J. (2022). Analysis of photosynthetic ability and related physiological traits in nodal leaves of grape. Sci. Hortic..

[172] Zheng H.-J., Wang X., Ma W.-F., Gou H.-M., Liang G.-P., Mao J. (2025). Temporal variations in photosynthesis and leaf element contents of ‘Marselan’ grapevines in response to foliar fertilizer application. Plants.

[173] Zinicovscaia I., Sturza R., Gurmeza I., Vergel K., Gundorina S., Duca G. (2019). Metal bioaccumulation in the soil–leaf–fruit system determined by neutron activation analysis. J. Food Meas. Charact..

[174] Zufferey V., Spring J.-L., Verdenal T., Dienes A., Belcher S., Lorenzini F., Koestel C., Rösti J., Gindro K., Spangenberg J. (2017). The influence of water stress on plant hydraulics, gas exchange, berry composition and quality of Pinot Noir wines in Switzerland. OENO One.

[175] Cohen J. (1988). Statistical Power Analysis for the Behavioral Sciences.

