# Supplementary material for: Ionome Dynamics in Grapevine Leaves
Source: Plants (Basel). 2026 Jun 30;15(13):2021. doi: 10.3390/plants15132021 (PMC13364328; doi:10.3390/plants15132021)
Supplement: Supplementary file 1 [file plants-15-02021-s001.zip › Supplementary tables and figures.pdf]

Ionome Dynamics in Grapevine Leaves

Jozef Kováčik [1*], Marek Vydra [2], Lenka Husáková [3], Martina Piroutková [3], Sławomir Dresler [4,5], Martin Dekan [6], František Duchoň [6]

[1] Department of Biology, Faculty of Education, University of Trnava, Priemyselná 4, 918 43 Trnava, Slovak Republic
[2] Department of Biology and Ecology, Faculty of Natural Sciences, Matej Bel University in Banská Bystrica, Tajovského 40, 974 01 Banská Bystrica, Slovak Republic
[3] Department of Analytical Chemistry, Faculty of Chemical Technology, University of Pardubice, Studentská 573 HB/D, 532 10 Pardubice, Czech Republic
[4] Department of Analytical Chemistry, Medical University of Lublin, 4A Chodzki St., 20-093 Lublin, Poland
[5] Department of Plant Physiology and Biophysics, Institute of Biological Sciences, Faculty of Biology and Biotechnology, Maria Curie-Skłodowska University, 19 Akademicka St., 20-033 Lublin, Poland
[6] Faculty of Electrical Engineering and Information Technology, Slovak University of Technology in Bratislava, Ilkovičova 2961, 841 04 Bratislava, Slovak Republic

* Correspondence: jozkovacik@yahoo.com (J.K.); Tel.: +421-33-5939-519
https://orcid.org/0000-0002-5701-0598

Table S1. Terms used in database search (Scopus and Web of Science). To ensure that the search results contain the key terms, the authors used Boolean operators: "OR" to unite multiple terms within a topic and "AND" to join them.

**Topic**	**Search terms**
Plant species	"*Vitis*" or "grape" or "vine"
Plant organ	"leaf" or "leaves"
Parameter	"metal" or "element" or "nutrient"

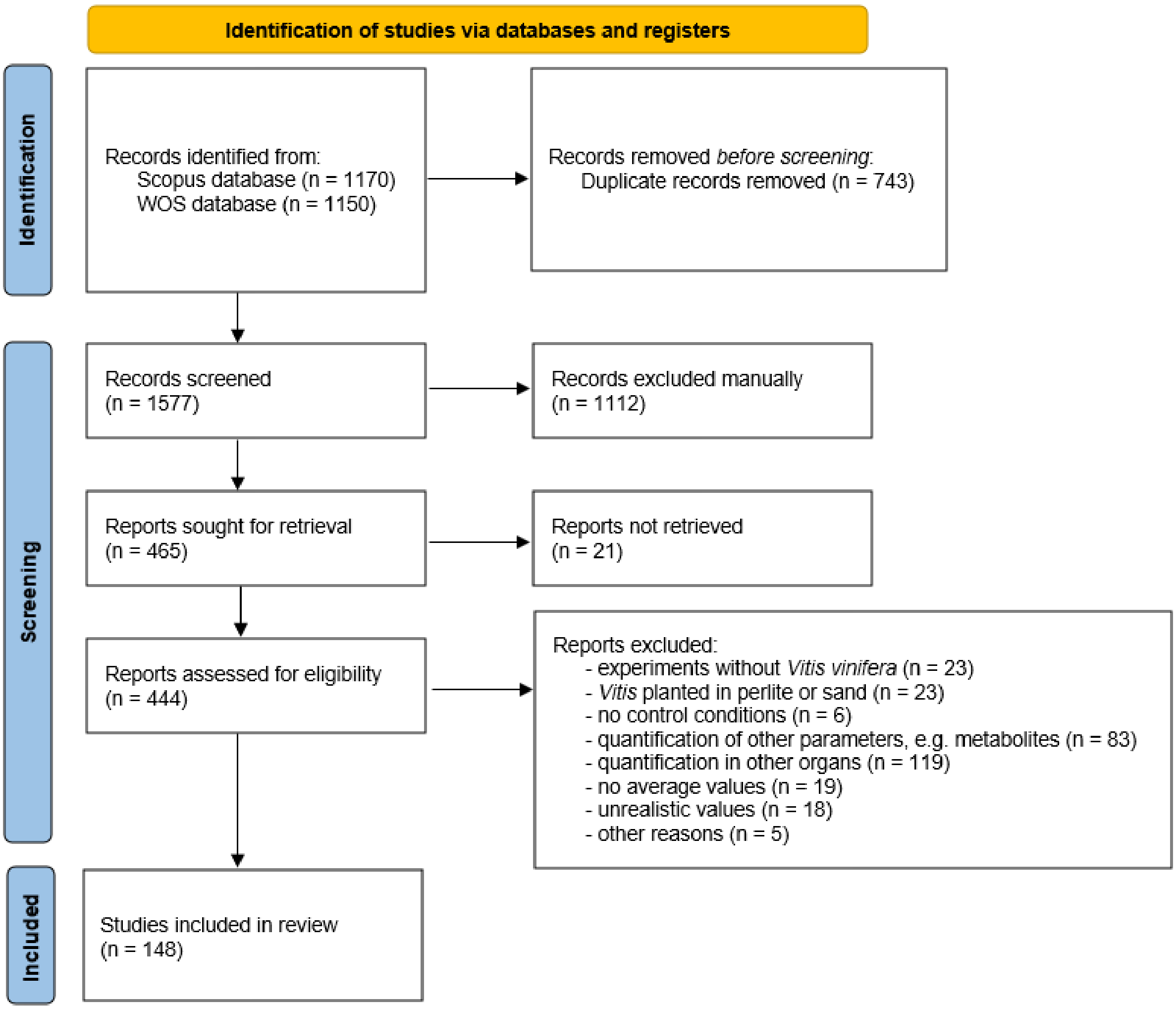

Figure S1. Methodology for selecting appropriate data from all studies published in the Scopus and Web of Science databases (using keywords mentioned in Table S1). From the primary dataset (1577 papers), 1112 were excluded based on the review of the paper title and abstract. The selection from 444 papers was carried out based on the manual review of the full text and the stated reasons for exclusion (e.g. by the term "unrealistic values" we mean mainly the concentration of trace elements/heavy metals at the level of mg element/g dry matter in control plants under standard conditions such as common agricultural soil or cultivation substrate). Diagram was created using the template by Page et al. (2021): https://www.bmj.com/concentration/372/bmj.n71

Table S2. Criteria for classifying selected data into specific categories of the investigated factors. The phenological stage was set according to the BBCH scale code for grapes (https://web.archive.org/web/20181015152947/https://ojs.openagrar.de/index.php/BBCH/article/view/515) and berry color/chlorotype according to VIVC (https://www.vivc.de/).

factor	category	criteria for inclusion
world area	Asia	*Vitis vinifera* was cultivated in Asian countries.
	Europe	*Vitis vinifera* was cultivated in European countries or the European part of Turkey.
	other continents	*Vitis vinifera* was cultivated in African and American countries.
stage of phenological development	flowering	The leaves were collected before or during flowering (BBCH scale code 69 or less). When a publication reported only the month of leaf harvest, we classified samples harvested in June or earlier into this category (only for Europe).
	berry set/green berries	The leaves were collected during fruit development and berry ripening (BBCH scale code: 71 to 79). When a publication reported only the month of leaf harvest, we classified samples harvested in July into this category (only for Europe).
	veraison	The leaves were collected during fruit development and berry ripening (BBCH scale code: 81 to 89). When a publication reported only the month of leaf harvest, we classified samples harvested in August into this category (only for Europe).
	harvest	The leaves were collected during or after harvest (BBCH scale code: 89 or more). When a publication reported only the month of leaf harvest, we classified samples harvested in September or later into this category (only for Europe).
berry color	white	Berry color of the cultivar specified as blanc in the *Vitis* International Variety Catalogue (VIVC).
	red	Berry color of the cultivar specified as noir, rouge or rose in the *Vitis* International Variety Catalogue (VIVC).
	np	Berry color of the cultivar is not provided in the *Vitis* International Variety Catalogue (VIVC).
chlorotype	a	Chlorotype of the cultivar specified as A in the *Vitis* International Variety Catalogue (VIVC).
	b	Chlorotype of the cultivar specified as B in the *Vitis* International Variety Catalogue (VIVC).
	c	Chlorotype of the cultivar specified as C in the *Vitis* International Variety Catalogue (VIVC).
	d	Chlorotype of the cultivar specified as D in the *Vitis* International Variety Catalogue (VIVC).
	np	Chlorotype of the cultivar is not provided in the *Vitis* International Variety Catalogue (VIVC).

Table S3-A. Operating conditions of the Agilent 7900 ICP-MS.

Parameter	Setting		
**ICP**			
Plasma mode	General purpose		
Rf power (27 MHz) (W)	1550		
Sampling depth (mm)	10		
Plasma gas flow (L/min)	15		
Auxiliary gas flow (L/min)	0.9		
Nebulizer gas flow (L/min)	1.05		
Nebulizer pump (rps)	0.1		
Spray chamber temperature (°C)	2		
**Mass spectrometer**	**No gas mode**	**He mode**	**HEHe mode** [a]
Extract 1 (V)	0	0	0
Extract 2 (V)	-250	-220	-225
Omega bias (V)	-90	-85	-105
Omega lens (V)	9.6	9	8.5
Cell entrance	-30	-40	-140
Cell exit	-50	-60	-150
Deflect (V)	12	1.6	-60
Plate bias	-35	-60	-150
Helium flow (mL/min)	0	6	10
OctP bias	-8	-18	-100
OctP RF	140	200	200
Energy discrimination (V)	5	5	9
Number of elements	16 [b]	12 [c]	5 [d]
**Acquisition**			
Points per peak	1		
Replicates	3		
Total acquisition time (s)	52		

[a] HEHe mode - high energy helium mode.

Monitored isotopes (integration time): [b)] (No gas mode) ^{7}Li (0.1 s), ^{9}Be (0.3 s), ^{11}B, ^{24}Mg, ^{66}Zn, ^{85}Rb, ^{88}Sr, ^{90}Zr, ^{95}Mo, ^{103}Rh, ^{111}Cd, ^{118}Sn, ^{121}Sb, ^{133}Cs, ^{138}Ba, $^{206+207+208}$Pb (all 0.1 s); [c)] (He mode) ^{23}Na (0.3 s), ^{27}Al (0.1 s), ^{39}K, ^{44}Ca (both 0.3 s), ^{51}V (1 s), ^{52}Cr, ^{55}Mn, ^{56}Fe, ^{59}Co, ^{60}Ni, ^{63}Cu, ^{103}Rh (all 0.3 s); [d)] (HE He mode) ^{31}P (0.1 s), ^{47}Ti (0.3 s), ^{75}As, ^{78}Se (both 1 s), ^{103}Rh (0.3 s).

Table S3-B. Method detection limits (MDL) [a] and method limits of quantification (MLOQ)[a] (μg kg^{-1}), and normalized calibration slopes (NCS) (L mg^{-1}) for the determination of selected elements in *Vitis vinifera* leaves by Agilent 7900 ICP-MS using Rh as an internal standard.

Analyte	Cell mode	NCS	MDL	MLOQ	Analyte	Cell mode	NCS	MDL	MLOQ
$^{7}Li^{+}$	No gas	1.3×10^{-2}	0.56	1.86	$^{60}Ni^{+}$	He	4.5×10^{-3}	7.5	25
$^{9}Be^{+}$	No gas	3.5×10^{-3}	0.13	0.45	$^{63}Cu^{+}$	He	1.3×10^{-2}	2.9	9.6
$^{11}B^{+}$	No gas	2.2×10^{-3}	10	35	$^{66}Zn^{+}$	No gas	4.8×10^{-3}	15	49
$^{23}Na^{+}$	He	5.8×10^{-4}	200	665	$^{75}As^{+}$	HE He	4.2×10^{-4}	0.89	3.0
$^{24}Mg^{+}$	No gas	1.8×10^{-2}	4.68	16	$^{78}Se^{+}$	HE He	4.8×10^{-5}	7.8	26
$^{27}Al^{+}$	He	2.3×10^{-5}	230	768	$^{85}Rb^{+}$	No gas	4.4×10^{-2}	0.31	1.0
$^{31}P^{+}$	HE He	5.5×10^{-6}	9.4	31	$^{88}Sr^{+}$	No gas	4.4×10^{-2}	0.35	1.2
$^{39}K^{+}$	He	1.1×10^{-4}	1452	4841	$^{90}Zr^{+}$	No gas	2.9×10^{-2}	0.12	0.45
$^{44}Ca^{+}$	He	6.6×10^{-6}	879	2928	$^{95}Mo^{+}$	No gas	8.4×10^{-3}	0.26	0.86
$^{47}Ti^{+}$	HE He	7.4×10^{-5}	17	58	$^{111}Cd^{+}$	No gas	6.0×10^{-3}	0.24	0.81
$^{51}V^{+}$	He	3.8×10^{-3}	0.14	0.47	$^{118}Sn^{+}$	No gas	1.4×10^{-2}	0.16	0.53
$^{52}Cr^{+}$	He	5.9×10^{-3}	4.6	15	$^{121}Sb^{+}$	No gas	1.9×10^{-2}	0.12	0.38
$^{55}Mn^{+}$	He	1.4×10^{-3}	2.2	7.2	$^{133}Cs^{+}$	No gas	6.7×10^{-2}	0.11	0.37
$^{56}Fe^{+}$	He	4.3×10^{-3}	5.4	18	$^{138}Ba^{+}$	No gas	4.4×10^{-2}	0.32	1.1
$^{59}Co^{+}$	He	1.4×10^{-2}	0.12	0.39	Pb [b]	No gas	5.5×10^{-2}	0.27	0.89

[a] Values were calculated assuming a sample mass of 0.100 g.

[b] Pb was quantified as the sum of the three most abundant isotopes ($^{206}Pb^{+}$, $^{207}Pb^{+}$, and $^{208}Pb^{+}$).

Supplementary Table S3-C. Comparison of measured and certified concentrations in selected certified reference materials (CRMs), including recoveries (R) and intra-day and inter-day relative standard deviations (RSD).

Analyte	Reference sample	Declared (mg kg^{-1})	Found[a] (mg kg^{-1})	R[b] (%)	RSD (%)	
					Intra	Inter
Li	GBW 10052 Green Tea	0.520 ± 0.04	0.448 ± 0.022	86	0.19	2.56
	GBW 07603 Bush Leaves	2.6 ± 0.4	2.72 ± 0.16	105	0.06	2.87
	NIST SRM 2702 Marine Sediment	78.2	73.6 ± 1.7	94	1.13	1.01
Be	GBW 10052 Green Tea	25 ± 3[c]	21.2 ± 2.3[c]	85	5.34	1.42
	GBW 07603 Bush Leaves	51 ± 4[c]	46 ± 4[c]	90	0.24	4.37
	NIST SRM 2702 Marine Sediment	3	3 ± 0.1	100	2.01	1.36
B	CRM 12-2-04 Wheat Bread Flour	< 0.5	0.55 ± 0.12	109	10.9	6.74
	GBW 10052 Green Tea	14.1 ± 1.2	14.0 ± 0.5	100	0.08	1.65
	BCR 679 White Cabbage	27.7 ± 1.9	26.0 ± 1.1	94	0.07	2.18
	CRM 12-2-03 Lucerne	30	25.7 ± 1.0	86	0.16	1.99
	GBW 07603 Bush Leaves	38 ± 6	39.8 ± 2.5	105	0.03	3.13
Na	CRM 12-2-04 Wheat Bread Flour	20	18.4 ± 1.5	92	4.08	7.95
	CRM 12-2-03 Lucerne	474 ± 23	509 ± 55	107	0.16	5.44
	NIST SRM 1633b Coal Fly Ash	2010 ± 30	1598 ± 146	80	4.58	3.35
	NIST SRM 2702 Marine Sediment	6810 ± 200	5777 ± 86	85	0.74	1.59
	GBW 07603 Bush Leaves	19600 ± 1800	18850 ± 4026	96	0.18	10.7
Mg	CRM 12-2-04 Wheat Bread Flour	556 ± 29	510 ± 80	92	0.05	7.86
	BCR 679 White Cabbage	1362 ± 127	1210 ± 20	89	0.09	0.81
	GBW 10052 Green Tea	2200 ± 80	1773 ± 353	81	0.13	9.96
	CRM 12-2-03 Lucerne	3520 ± 125	3344 ± 135	95	0.07	2.01
	GBW 07603 Bush Leaves	4800 ± 400	4453 ± 712	93	0.11	7.99
	NIST SRM 1633b Coal Fly Ash	4820 ± 80	3848 ± 473	80	6.14	2.85
	NIST SRM 2702 Marine Sediment	9900 ± 740	10075 ± 353	102	1.75	2.21
Al	CRM 12-2-04 Wheat Bread Flour	3	3.23 ± 0.23	108	3.53	1.39
	CRM 12-2-03 Lucerne	330	334 ± 17	101	2.54	1.66
	NIST SRM 2702 Marine Sediment	84100 ± 2200	85272 ± 1475	101	0.86	3.87
	NIST SRM 1633b Coal Fly Ash	150500 ± 2700	124033 ± 17682	83	7.13	5.56
P	GBW 07603 Bush Leaves	1000 ± 40	928 ± 7.8	93	0.42	1.31
	NIST SRM 2702 Marine Sediment	1552 ± 66	1560 ± 29	100	0.94	4.02
	NIST SRM 1633b Coal Fly Ash	2300	1977 ± 27	86	0.68	0.69
	GBW 10052 Green Tea	2800 ± 100	2344 ± 411	84	0.02	8.76
	CRM 12-2-03 Lucerne	3030 ± 90	2810 ± 131	93	0.13	2.33
	BCR 679 White Cabbage	3307 ± 241	3173 ± 66	96	0.03	1.04
K	GBW 07603 Bush Leaves	9200 ± 1000	9230 ± 1732	100	0.07	9.38
	GBW 10052 Green Tea	15500 ± 700	13220 ± 2300	85	0.04	8.70
	CRM 12-2-03 Lucerne	18700 ± 650	18400 ± 821	99	0.11	2.23
	NIST SRM 2702 Marine Sediment	20540 ± 720	17222 ± 762	84	2.21	4.64
Ca	CRM 12-2-04 Wheat Bread Flour	292 ± 30	274 ± 39	94	0.27	7.12
	NIST SRM 2702 Marine Sediment	3430 ± 240	3387 ± 351	99	5.19	8.83
	BCR 679 White Cabbage	7768 ± 655	7570 ± 336	97	0.14	2.22
	GBW 10052 Green Tea	12100 ± 300	9910 ± 1840	82	0.09	9.22
	NIST SRM 1633b Coal Fly Ash	15100 ± 600	16623 ± 1345	110	4.05	1.47
	GBW 07603 Bush Leaves	16800 ± 1100	17150 ± 2840	102	0.13	8.27
	CRM 12-2-03 Lucerne	17500 ± 750	16910 ± 573	97	0.11	1.69
Ti	NIST SRM 1633b Coal Fly Ash	7910 ± 140	6335 ± 821	80	6.48	3.55
V	GBW 10052 Green Tea	0.6 ± 0.02	0.492 ± 0.032	82	0.08	3.30
	GBW 07603 Bush Leaves	2.4 ± 0.4	2.13 ± 0.16	89	0.14	3.80
	NIST SRM 1633b Coal Fly Ash	296 ± 3.6	261 ± 1.6	88	0.31	3.70
	NIST SRM 2702 Marine Sediment	358 ± 9.2	348 ± 39	97	5.58	1.45

Analyte	Reference sample	Declared (mg kg^{-1})	Found[a] (mg kg^{-1})	R[b] (%)	RSD (%)	
					Intra	Inter
Cr	BCR 679 White Cabbage	0.6 ± 0.1	0.57 ± 0.05	94	4.29	2.63
	CRM 12-2-03 Lucerne	0.900	0.84 ± 0.01	93	0.76	4.39
	GBW 10052 Green Tea	0.92 ± 0.2	0.774 ± 0.131	84	0.03	8.47
	NIST SRM 1633b Coal Fly Ash	198 ± 4.7	176 ± 3.3	89	0.94	5.17
	NIST SRM 2702 Marine Sediment	352 ± 22	331 ± 44	94	6.65	0.86
Mn	BCR 679 White Cabbage	13.3 ± 0.5	13.9 ± 0.69	105	0.09	2.46
	CRM 12-2-04 Wheat Bread Flour	22.6 ± 1.1	23.0 ± 0.45	102	0.07	0.98
	CRM 12-2-03 Lucerne	34.2 ± 1.2	37.3 ± 4.6	109	0.13	6.23
	GBW 07603 Bush Leaves	61 ± 5	61 ± 0.4	100	0.30	0.85
	NIST SRM 1633b Coal Fly Ash	132 ± 1.7	114 ± 0.5	86	0.24	3.02
	GBW 10052 Green Tea	1170 ± 60	1183 ± 41	101	0.09	1.73
	NIST SRM 2702 Marine Sediment	1757 ± 58	1626 ± 48	93	1.46	3.46
Fe	CRM 12-2-04 Wheat Bread Flour	23.8 ± 1.5	24.5 ± 0.36	103	0.06	0.74
	BCR 679 White Cabbage	55 ± 2.5	53.2 ± 0.89	97	0.10	0.84
	GBW 10052 Green Tea	322 ± 23	296 ± 5.3	92	0.08	0.89
	CRM 12-2-03 Lucerne	355 ± 18	355 ± 40	100	0.06	5.67
	GBW 07603 Bush Leaves	1070 ± 57	1030 ±46	96	0.05	1.10
	NIST SRM 2702 Marine Sediment	74000	71398 ± 7014	96	4.91	0.89
	NIST SRM 1633b Coal Fly Ash	77800 ± 2300	68714 ± 431	88	0.31	3.35
Co	GBW 10052 Green Tea	0.3 ± 0.02	0.263 ± 0.004	88	0.09	0.69
	GBW 07603 Bush Leaves	0.41 ± 0.05	0.460 ± 0.045	112	0.04	4.93
	NIST SRM 2702 Marine Sediment	27.8 ± 0.58	26.4 ± 1.5	95	2.75	1.12
	NIST SRM 1633b Coal Fly Ash	50	44.8 ± 2.4	90	2.66	3.14
Ni	GBW 07603 Bush Leaves	1.7 ± 0.3	1.47 ± 0.074	86	0.01	2.53
	GBW 10052 Green Tea	5.4 ± 0.4	5.58 ± 0.097	103	0.08	0.87
	BCR 679 White Cabbage	27 ± 0.8	26.8 ± 0.30	99	0.03	0.55
	NIST SRM 2702 Marine Sediment	75.4 ± 1.5	73.3 ± 1.5	97	1.02	1.38
	NIST SRM 1633b Coal Fly Ash	121 ± 1.8	111 ± 3.3	92	1.50	2.57
Cu	CRM 12-2-04 Wheat Bread Flour	2.77 ± 0.03	2.82 ± 0.04	102	0.03	7.59
	BCR 679 White Cabbage	2.89 ± 0.12	2.72 ± 0.039	94	0.01	0.73
	GBW 07603 Bush Leaves	6.6 ± 0.8	6.3 ± 0.59	96	0.07	4.64
	CRM 12-2-03 Lucerne	11.7 ± 0.75	11.7 ± 0.36	100	0.03	1.56
	GBW 10052 Green Tea	24 ± 1	23.2 ± 0.91	97	0.14	1.97
	NIST SRM 1633b Coal Fly Ash	113 ± 2.6	101 ± 3.7	90	1.84	2.86
	NIST SRM 2702 Marine Sediment	118 ± 5.6	112 ± 1.5	95	0.66	0.41
Zn	CRM 12-2-04 Wheat Bread Flour	17.9 ± 0.75	18.8 ± 0.96	105	0.04	2.56
	GBW 10052 Green Tea	35 ± 2	35.6 ± 1.1	102	0.04	1.54
	GBW 07603 Bush Leaves	55 ± 4	59.9 ± 3.7	109	0.05	3.10
	BCR 679 White Cabbage	79.7 ± 2.7	82.0 ± 1.4	103	0.04	0.83
	NIST SRM 1633b Coal Fly Ash	210	211 ± 6.6	101	1.55	1.43
	NIST SRM 2702 Marine Sediment	486 ± 4.2	488 ± 1.7	100	1.76	0.79
As	BCR 679 White Cabbage	6.7[c]	6.7 ± 2.6[c]	100	0.24	10.8
	CRM 12-2-04 Wheat Bread Flour	17 ± 4.6[c]	15.9 ± 3.3[c]	93	0.66	10.3
	CRM 12-2-03 Lucerne	0.262 ± 0.020	0.258 ± 0.031	98	0.39	5.94
	GBW 10052 Green Tea	0.27 ± 0.05	0.265 ± 0.030	98	0.12	5.65
	GBW 07603 Bush Leaves	1.25 ± 0.15	1.33 ± 0.20	107	0.03	7.36
	NIST SRM 2702 Marine Sediment	45.3 ± 1.8	46.2 ± 4.1	102	4.47	0.53
	NIST SRM 1633b Coal Fly Ash	136 ± 2.6	159 ± 1.5	117	0.48	9.11
Se	CRM 12-2-04 Wheat Bread Flour	40[c]	40.4 ± 2.9[c]	101	3.60	11.9
	GBW 10052 Green Tea	0.10 ± 0.03	0.100 ± 0.01	100	0.96	5.45

Analyte	Reference sample	Declared (mg kg^{-1})	Found[a] (mg kg^{-1})	R[b] (%)	RSD (%)	
					Intra	Inter
Rb	CRM 12-2-04 Wheat Bread Flour	1.5	1.41 ± 0.030	94	0.02	1.08
	CRM 12-2-03 Lucerne	16.1 ± 2.2	14.6 ± 0.89	91	0.16	3.07
	GBW 10052 Green Tea	89 ± 9	85.3 ± 3.6	96	0.05	2.13
	NIST SRM 2702 Marine Sediment	128 ± 8.8	119 ± 2.6	93	1.11	1.12
	NIST SRM 1633b Coal Fly Ash	140	118 ± 4	85	1.72	3.54
Sr	CRM 12-2-04 Wheat Bread Flour	1.53 ± 0.16	1.50 ± 0.03	98	0.05	1.16
	BCR 679 White Cabbage	11.8 ± 0.4	11.5 ± 0.20	97	0.04	0.87
	GBW 10052 Green Tea	36 ± 2	33.8 ± 1.2	94	0.07	1.83
	NIST SRM 2702 Marine Sediment	120 ± 3	117 ± 1.7	97	0.73	0.90
	GBW 07603 Bush Leaves	246 ± 16	260 ± 17	106	0.04	3.44
	NIST SRM 1633b Coal Fly Ash	1041 ± 14	960 ± 11	92	0.56	1.15
Mo	GBW 10052 Green Tea	0.110 ± 0.02	0.090 ± 0.014	82	0.09	8.05
	CRM 12-2-04 Wheat Bread Flour	0.2	0.206 ± 0.010	103	0.08	2.32
	CRM 12-2-03 Lucerne	0.200	0.183 ± 0.008	92	0.02	2.12
	GBW 07603 Bush Leaves	0.280 ± 0.05	0.280 ± 0.012	100	0.14	7.86
	NIST SRM 2702 Marine Sediment	10.8 ± 1.6	9.47 ± 0.33	88	1.75	1.81
	BCR 679 White Cabbage	14.8 ± 0.5	14.9 ± 0.20	101	0.03	0.66
Cd	CRM 12-2-04 Wheat Bread Flour	41.5 ± 3.2[c]	37.9 ± 3.7[c]	91	0.58	4.88
	GBW 10052 Green Tea	76 ± 4[c]	74.9 ± 8.4[c]	99	0.15	5.61
	CRM 12-2-03 Lucerne	0.136 ± 0.007	0.128 ± 0.006	94	0.25	2.29
	NIST SRM 1633b Coal Fly Ash	0.784 ± 0.006	0.867 ± 0.059	111	3.40	2.46
	NIST SRM 2702 Marine Sediment	0.817 ± 0.011	0.819 ± 0.007	100	0.45	0.42
	BCR 679 White Cabbage	1.66 ± 0.07	1.66 ± 0.04	100	0.04	1.22
Sn	GBW 10052 Green Tea	170[c]	169 ± 5.9[c]	100	1.74	6.76
	NIST SRM 2702 Marine Sediment	31.6 ± 2.4	31.5 ± 0.7	100	1.18	0.14
Sb	GBW 10052 Green Tea	52[c]	52.2 ± 1.5[c]	100	1.47	8.77
	GBW 07603 Bush Leaves	95 ± 14[c]	88.9 ± 0.7[c]	92	0.38	8.14
	NIST SRM 2702 Marine Sediment	5.6 ± 0.24	4.5 ± 0.15	81	1.69	2.45
	BCR 679 White Cabbage	20.6 ± 2.6[c]	17.4 ± 4.6[c]	84	0.33	2.26
Cs	CRM 12-2-04 Wheat Bread Flour	5[c]	4.4 ± 0.7[c]	87	7.96	0.63
	CRM 12-2-03 Lucerne	90[c]	85 ± 6.0[c]	94	0.02	3.58
	GBW 07603 Bush Leaves	0.270 ± 0.020	0.256 ± 0.014	95	0.03	2.75
	GBW 10052 Green Tea	0.580 ± 0.03	0.552 ± 0.027	95	0.03	2.47
	NIST SRM 2702 Marine Sediment	7.1	7.1 ± 0.1	100	0.87	1.38
	NIST SRM 1633b Coal Fly Ash	11	10.3 ± 0.13	94	0.87	3.98
Ba	CRM 12-2-04 Wheat Bread Flour	1.5	1.51 ± 0.012	101	0.42	0.41
	BCR 679 White Cabbage	10.3 ± 0.6	10.6 ± 0.7	103	0.06	3.30
	GBW 07603 Bush Leaves	18 ± 2	17.5 ± 0.52	97	0.07	1.49
	CRM 12-2-03 Lucerne	23.4 ± 2.1	24.4 ± 1.8	104	3.75	11.4
	GBW 10052 Green Tea	41 ± 4	41.5 ± 1.09	101	0.05	1.31
	NIST SRM 2702 Marine Sediment	397 ± 3.2	399 ± 12	101	1.44	0.38
	NIST SRM 1633b Coal Fly Ash	709 ± 28	696 ± 21	98	1.54	2.64
Pb[d]	CRM 12-2-04 Wheat Bread Flour	41 ± 7.8[c]	39 ± 7.2[c]	93	0.31	9.34
	GBW 10052 Green Tea	1.6 ± 0.2	1.50 ± 0.080	94	0.11	2.67
	CRM 12-2-03 Lucerne	1.84 ± 0.17	1.9 ± 0.05	103	1.32	1.60
	GBW 07603 Bush Leaves	47 ± 3	52.3 ± 1.9	111	0.11	1.86
	NIST SRM 1633b Coal Fly Ash	68.2 ± 1.1	69.3 ± 5.7	102	4.11	2.15
	NIST SRM 2702 Marine Sediment	133 ± 1.1	133 ± 4.2	100	1.58	2.82

[a] Mean ± 2 S.D. (n = 3)

[b] Recovery (%) = (found value/certified value)×100

[c] Units: µg kg^{-1}

[d] Pb was quantified as the sum of the three most abundant isotopes ($^{206}Pb^+$, $^{207}Pb^+$, and $^{208}Pb^+$).

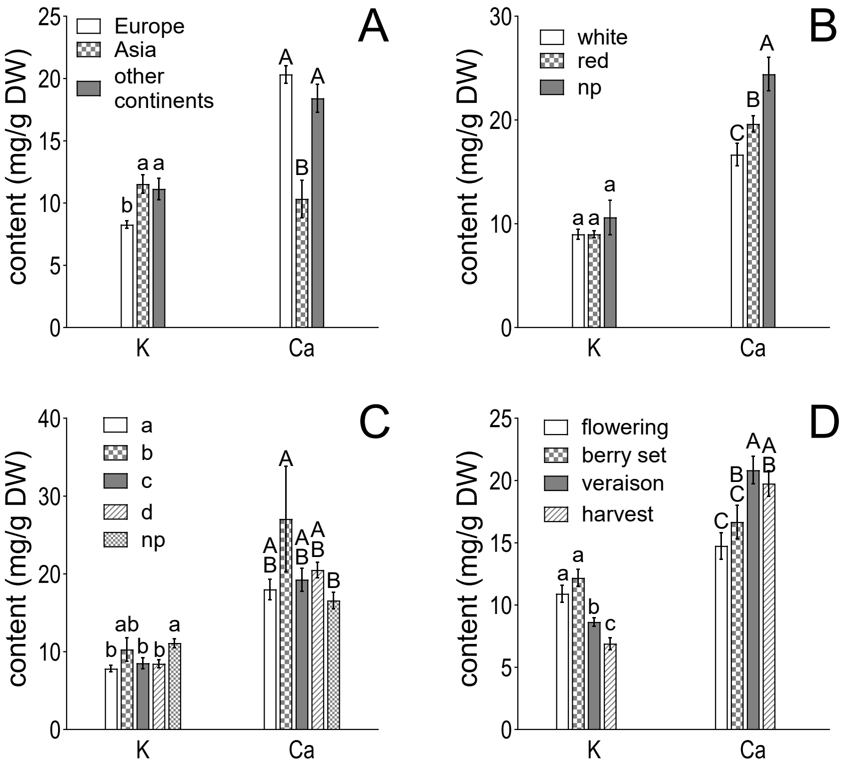

Figure S2. K and Ca concentration in *Vitis vinifera* leaves with respect to world area (A), berry color (B), chlorotype (C), and stage of phenological development (D). Data are means ± SE. Columns followed by the same letter(s) are not significantly different according to the Bonferroni post-hoc test ($p > 0.05$). The detailed descriptive statistics of individual groups within the examined factors are presented in the supplementary Tables S4 and S5.

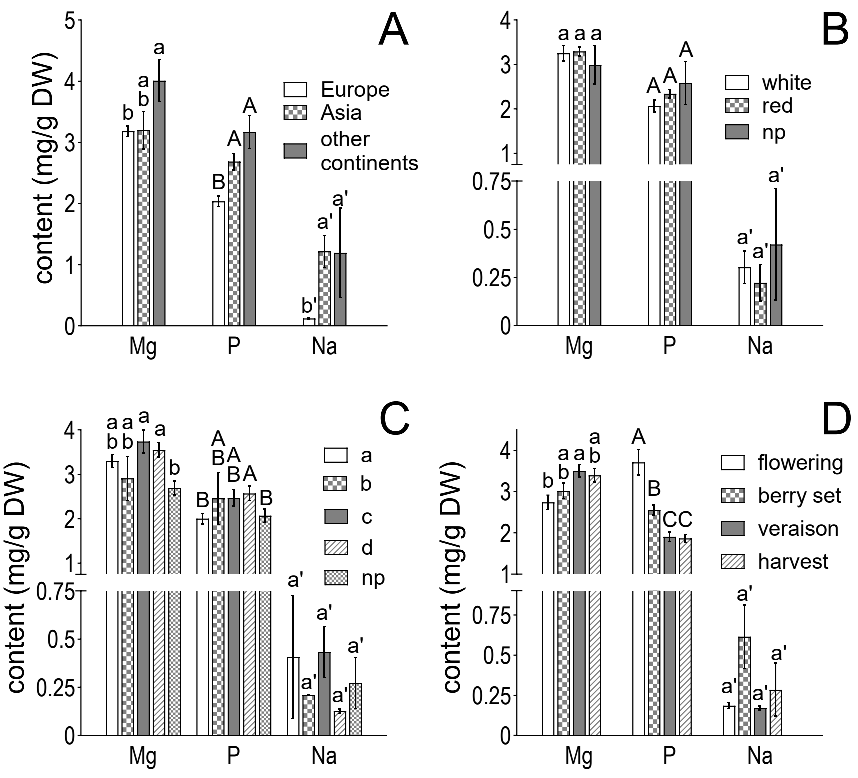

Figure S3. Mg, P and Na concentration in *Vitis vinifera* leaves with respect to world area (A), berry color (B), chlorotype (C), and stage of phenological development (D). Data are means ± SE. Columns followed by the same letter(s) are not significantly different according to the Bonferroni post-hoc test ($p > 0.05$). The detailed descriptive statistics of individual groups within the examined factors are presented in the supplementary Tables S4 and S5.

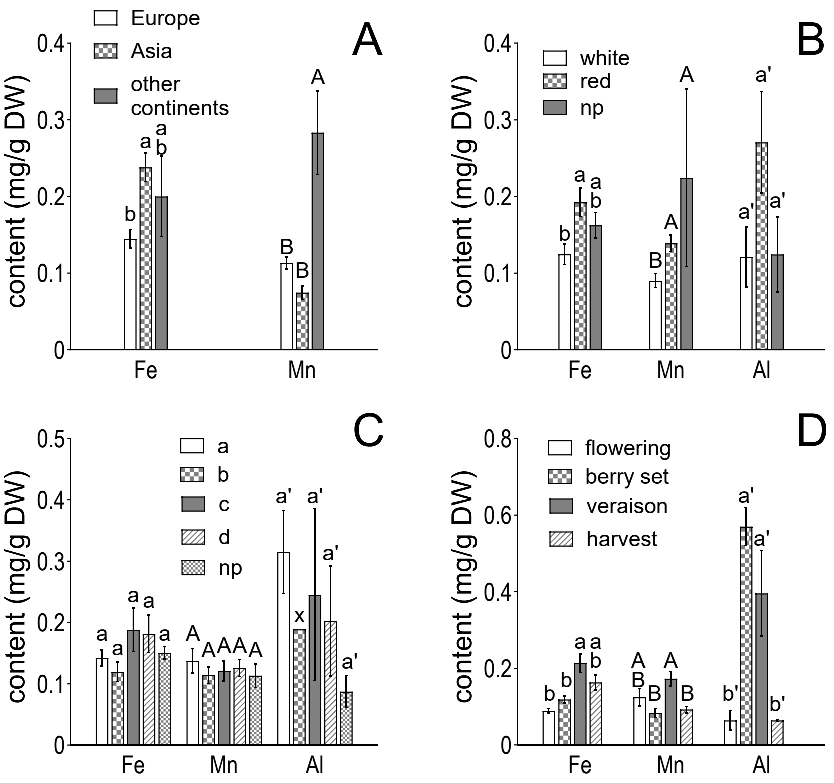

Figure S4. Fe, Mn, and Al concentration in *Vitis vinifera* leaves with respect to world area (A), berry color (B), chlorotype (C), and stage of phenological development (D). Data are means ± SE. Columns followed by the same letter(s) are not significantly different according to the Bonferroni post-hoc test ($p > 0.05$). The column marked with "x" was excluded from statistical analyses due to an insufficient number of observations (the value is shown for comparison only). A comparison of Al concentration with respect to continents was not performed because there were not enough observations from Asia or other continents available. The detailed descriptive statistics of individual groups within the examined factors are presented in the supplementary Tables S4 and S5.

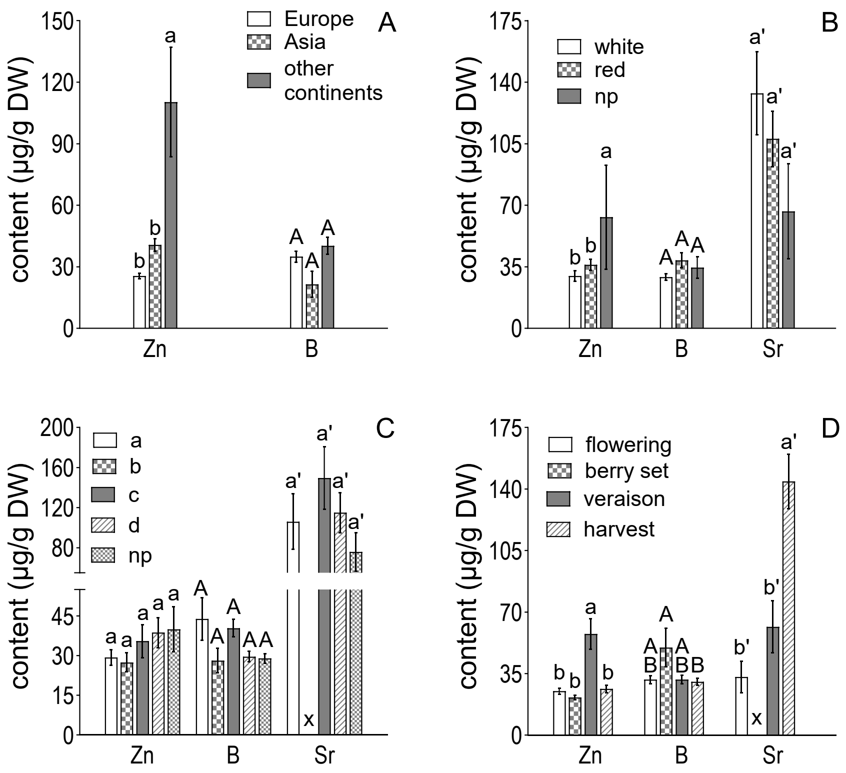

Figure S5. Zn, B, and Sr concentration in *Vitis vinifera* leaves with respect to world area (A), berry color (B), chlorotype (C), and stage of phenological development (D). Data are means ± SE. Columns followed by the same letter(s) are not significantly different according to the Bonferroni post-hoc test ($p > 0.05$). The column marked with "x" was excluded from statistical analyses due to a lack of data. Sr concentration comparison with respect to world area was not performed because there is not enough data available from Asia or other continents. The detailed descriptive statistics of individual groups within the examined factors are presented in the supplementary Tables S4 and S5.

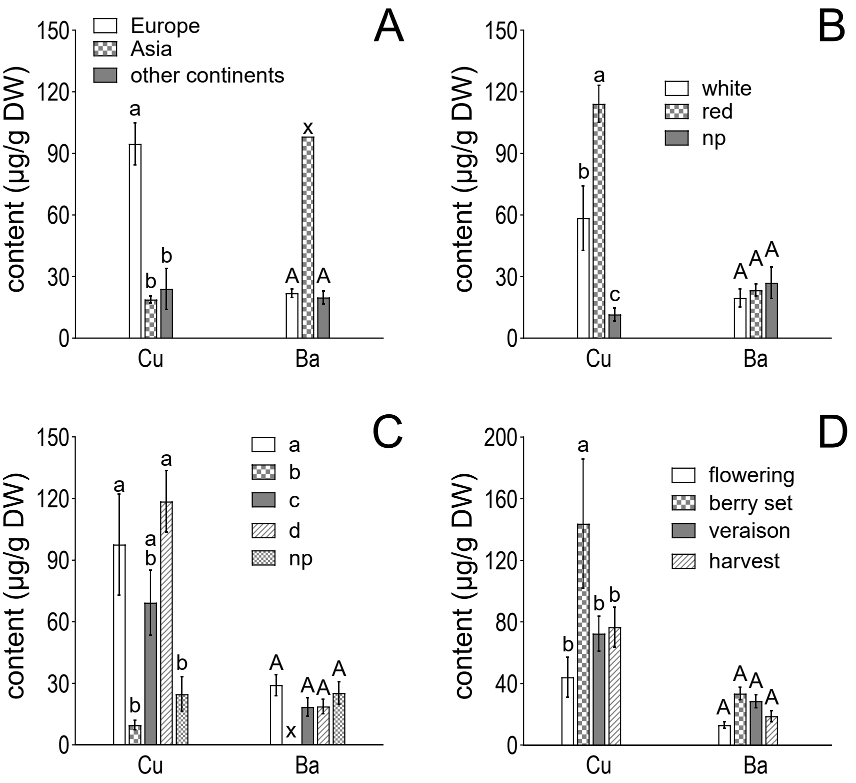

Figure S6. Cu and Ba concentration in *Vitis vinifera* leaves with respect to world area (A), berry color (B), chlorotype (C), and stage of phenological development (D). Data are means ± SE. Columns followed by the same letter(s) are not significantly different according to the Bonferroni post-hoc test ($p > 0.05$). The column marked with "x" was excluded from statistical analyses due to a lack of data or an insufficient number of observations. The detailed descriptive statistics of individual groups within the examined factors are presented in the supplementary Tables S4 and S5.

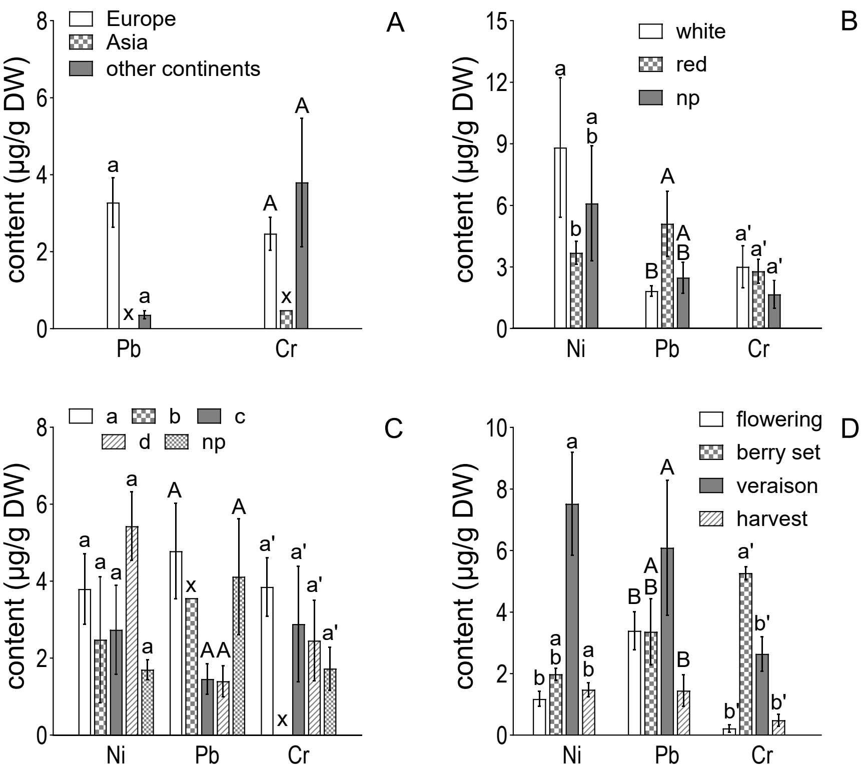

Figure S7. Ni, Pb and Cr concentration in *Vitis vinifera* leaves with respect to world area (A), berry color (B), chlorotype (C), and stage of phenological development (D). Data are means ± SE. Columns followed by the same letter(s) are not significantly different according to Bonferroni post-hoc test ($p > 0.05$). Column marked with "x" was excluded from statistical analyses due to no data/an insufficient number of observations. Ni concentration comparison with respect to world area was not performed because there were not enough observations from Asia or other continents. The detailed descriptive statistics of individual groups within the examined factors are presented in the supplementary Tables S4 and S5.

Table S4. Descriptive statistics of the concentration of analyzed elements in specific groups within examined factors (world area, berry color, chlorotype and stage of phenological development). N – number of values for a given element, SD – standard deviation, SE – standard error. In this way, only elements that had a sufficient number of observations for group comparison (using GLMs) were processed.

factor	group	N	Mean	SD	SE	lower 95% CI	upper 95% CI
			**K (mg/g)**				
area	Europe	262	8.27	4.82	0.30	7.68	8.85
	Asia	49	11.53	5.05	0.72	10.08	12.98
	other	43	11.14	5.67	0.86	9.40	12.89
berry color	white	130	8.99	5.43	0.48	8.05	9.94
	red	207	8.98	4.77	0.33	8.33	9.64
	np	17	10.62	6.84	1.66	7.10	14.14
chlorotype	a	95	7.84	4.17	0.43	6.99	8.69
	b	10	10.30	4.79	1.52	6.87	13.73
	c	57	8.52	5.49	0.73	7.06	9.98
	d	97	8.45	4.77	0.48	7.49	9.41
	np	95	11.12	5.60	0.57	9.98	12.26
stage of phenological development	flowering	41	10.91	4.30	0.67	9.55	12.27
	berry set	70	12.18	5.74	0.69	10.81	13.55
	veraison	134	8.64	4.03	0.35	7.95	9.33
	harvest	109	6.90	5.05	0.48	5.94	7.86
			**Ca (mg/g)**				
area	Europe	221	20.33	10.45	0.70	18.95	21.72
	Asia	37	10.34	9.09	1.49	7.31	13.37
	other	32	18.41	6.40	1.13	16.10	20.71
berry color	white	98	16.67	10.71	1.08	14.53	18.82
	red	179	19.63	10.31	0.77	18.11	21.15
	np	13	24.43	5.80	1.61	20.92	27.93
chlorotype	a	73	18.01	11.14	1.30	15.42	20.61

	b	8	27.05	19.23	6.80	10.98	43.13
	c	52	19.26	10.54	1.46	16.33	22.20
	d	84	20.50	9.28	1.01	18.48	22.51
	np	73	16.58	9.05	1.06	14.47	18.69
stage of phenological development	flowering	40	14.76	6.71	1.06	12.61	16.90
	berry set	61	16.67	10.50	1.34	13.98	19.36
	veraison	115	20.84	11.81	1.10	18.66	23.02
	harvest	74	19.75	8.82	1.03	17.71	21.80
**Mg (mg/g)**							
area	Europe	221	3.18	1.33	0.09	3.01	3.36
	Asia	38	3.20	1.89	0.31	2.58	3.82
	other	29	4.01	1.87	0.35	3.30	4.72
berry color	white	105	3.25	1.77	0.17	2.91	3.60
	red	172	3.30	1.30	0.10	3.10	3.49
	np	11	2.99	1.43	0.43	2.03	3.95
chlorotype	a	70	3.30	1.20	0.14	3.01	3.59
	b	8	2.91	1.41	0.50	1.74	4.09
	c	48	3.74	1.78	0.26	3.22	4.26
	d	83	3.55	1.48	0.16	3.23	3.87
	np	79	2.69	1.39	0.16	2.38	3.00
stage of phenological development	flowering	37	2.74	1.05	0.17	2.39	3.09
	berry set	65	3.02	1.47	0.18	2.65	3.39
	veraison	113	3.50	1.62	0.15	3.20	3.80
	harvest	73	3.40	1.41	0.16	3.07	3.72
**P (mg/g)**							
area	Europe	205	2.04	1.23	0.09	1.87	2.21
	Asia	42	2.69	0.87	0.13	2.41	2.96
	other	24	3.17	1.32	0.27	2.61	3.73
Berry color	white	105	2.06	1.36	0.13	1.80	2.33
	red	160	2.34	1.16	0.09	2.16	2.52
	np	6	2.59	1.19	0.49	1.34	3.83
chlorotype	a	85	2.00	1.09	0.12	1.77	2.24
	b	9	2.46	1.75	0.58	1.11	3.81

	c	44	2.47	1.25	0.19	2.09	2.85
	d	60	2.57	1.26	0.16	2.25	2.90
	np	73	2.07	1.27	0.15	1.77	2.37
stage of phenological development	flowering	27	3.71	1.62	0.31	3.07	4.35
	berry set	68	2.55	1.02	0.12	2.31	2.80
	veraison	101	1.91	1.18	0.12	1.67	2.14
	harvest	75	1.87	0.84	0.10	1.68	2.06
**Na (mg/g)**							
area	Europe	89	0.124	0.087	0.009	0.105	0.142
	Asia	6	1.220	0.641	0.262	0.547	1.893
	other	8	1.196	2.064	0.730	-0.530	2.922
berry color	white	35	0.303	0.495	0.084	0.133	0.473
	red	57	0.222	0.717	0.095	0.032	0.412
	np	11	0.422	0.961	0.290	-0.224	1.067
chlorotype	a	17	0.410	1.310	0.320	-0.268	1.083
	b	2	0.210	0.001	0.001	0.198	0.222
	c	21	0.430	0.610	0.130	0.157	0.710
	d	39	0.130	0.080	0.010	0.102	0.150
	np	24	0.270	0.650	0.130	-0.003	0.548
stage of phenological development	flowering	16	0.185	0.075	0.019	0.145	0.225
	harvest	38	0.285	1.016	0.165	-0.049	0.619
	veraison	36	0.171	0.080	0.013	0.144	0.198
	berry set	13	0.615	0.717	0.199	0.181	1.048
**Fe (mg/g)**							
area	Europe	231	0.145	0.184	0.012	0.121	0.169
	Asia	38	0.238	0.114	0.019	0.201	0.276
	other	29	0.200	0.283	0.053	0.093	0.308
berry color	white	123	0.125	0.145	0.013	0.099	0.151
	red	153	0.192	0.227	0.018	0.156	0.229
	np	22	0.163	0.078	0.017	0.128	0.197
chlorotype	a	63	0.142	0.105	0.013	0.116	0.169
	b	10	0.120	0.052	0.016	0.083	0.157
	c	49	0.188	0.249	0.036	0.116	0.259
	d	82	0.181	0.277	0.031	0.120	0.242
	np	94	0.150	0.097	0.010	0.130	0.170
	flowering	42	0.089	0.036	0.006	0.078	0.100

stage of phenological development	berry set	62	0.119	0.070	0.009	0.101	0.137
	veraison	111	0.214	0.256	0.024	0.166	0.262
	harvest	83	0.163	0.181	0.020	0.124	0.203
**Al (mg/g)**							
berry color	white	17	0.121	0.162	0.039	0.038	0.205
	red	36	0.271	0.398	0.066	0.136	0.405
	np	7	0.124	0.129	0.049	0.005	0.244
chlorotype	a	15	0.315	0.262	0.068	0.170	0.460
	b	1	0.189	-	-	-	-
	c	11	0.245	0.465	0.140	-0.067	0.557
	d	19	0.202	0.392	0.090	0.014	0.391
	np	14	0.087	0.097	0.026	0.031	0.143
stage of phenological development	flowering	4	0.064	0.052	0.026	-0.018	0.147
	berry set	5	0.570	0.110	0.049	0.433	0.707
	veraison	19	0.396	0.486	0.112	0.161	0.630
	harvest	32	0.064	0.016	0.003	0.058	0.070
**Mn (mg/g)**							
area	Europe	199	0.113	0.108	0.008	0.098	0.128
	Asia	38	0.075	0.054	0.009	0.057	0.092
	other	25	0.283	0.272	0.054	0.171	0.396
berry color	white	98	0.090	0.090	0.009	0.072	0.108
	red	154	0.139	0.133	0.011	0.118	0.160
	np	10	0.225	0.367	0.116	-0.038	0.487
chlorotype	a	62	0.138	0.158	0.020	0.097	0.178
	b	10	0.114	0.043	0.014	0.083	0.145
	c	47	0.121	0.111	0.016	0.089	0.154
	d	74	0.126	0.121	0.014	0.098	0.154
	np	69	0.113	0.161	0.019	0.075	0.152
stage of phenological development	flowering	39	0.125	0.139	0.022	0.080	0.170
	berry set	55	0.084	0.084	0.011	0.061	0.106
	veraison	93	0.173	0.182	0.019	0.135	0.210
	harvest	75	0.093	0.074	0.009	0.076	0.110
**Zn (μg/g)**							
area	Europe	229	25.51	20.50	1.35	22.84	28.18
	Asia	39	40.68	18.43	2.95	34.71	46.65
	other	30	110.31	146.32	26.71	55.67	164.94

berry color	white	111	29.75	31.94	3.03	23.74	35.76
	red	162	36.12	40.23	3.16	29.88	42.37
	np	25	63.30	147.98	29.60	2.22	124.38
chlorotype	a	68	29.32	24.07	2.92	23.49	35.14
	b	10	27.44	11.27	3.57	19.37	35.50
	c	50	35.49	44.19	6.25	22.93	48.05
	d	81	38.74	51.18	5.69	27.42	50.05
	np	89	39.97	80.51	8.53	23.01	56.92
stage of phenological development	flowering	44	25.04	11.24	1.69	21.62	28.45
	berry set	62	21.45	9.99	1.27	18.91	23.98
	veraison	104	57.63	88.20	8.65	40.47	74.78
	harvest	88	26.28	19.50	2.08	22.15	30.41
**B (µg/g)**							
area	Europe	153	34.97	34.15	2.76	29.52	40.43
	Asia	5	21.47	14.11	6.31	3.94	38.99
	other	11	40.24	13.86	4.18	30.93	49.55
berry color	white	63	29.18	15.15	1.91	25.37	33.00
	red	96	38.71	41.07	4.19	30.39	47.03
	np	10	34.55	19.23	6.08	20.79	48.30
chlorotype	a	49	43.90	56.54	8.08	27.66	60.14
	b	6	28.16	11.36	4.64	16.24	40.09
	c	22	40.42	15.32	3.27	33.63	47.21
	d	37	29.63	11.83	1.94	25.68	33.57
	np	55	29.00	13.13	1.77	25.45	32.55
stage of phenological development	flowering	25	31.61	11.35	2.27	26.92	36.29
	berry set	35	49.86	64.24	10.86	27.79	71.93
	veraison	49	31.58	16.91	2.42	26.72	36.43
	harvest	60	30.30	15.51	2.00	26.29	34.30
**Sr (µg/g)**							
berry color	white	16	133.71	94.12	23.53	83.56	183.87
	red	29	107.81	84.75	15.74	75.58	140.05
	np	8	66.55	76.39	27.01	2.69	130.42
chlorotype	a	10	106.00	87.76	27.75	43.22	168.78
	b	0	-	-	-	-	-
	c	11	149.54	103.91	31.33	79.73	219.34
	d	17	115.03	82.51	20.01	72.61	157.45

	np	15	75.87	74.45	19.22	34.64	117.10
stage of phenological development	flowering	4	33.13	17.98	8.99	4.52	61.73
	berry set	0	-	-	-	-	-
	veraison	17	61.58	61.19	14.84	30.12	93.04
	harvest	32	144.34	87.43	15.46	112.82	175.86
**Cu (µg/g)**							
area	Europe	157	94.60	128.47	10.25	74.34	114.85
	Asia	30	18.79	9.07	1.66	15.40	22.17
	other	23	24.00	47.79	9.96	3.33	44.66
berry color	white	83	58.52	143.68	15.77	82.77	145.51
	red	107	114.14	92.60	8.95	40.77	76.27
	np	20	11.58	14.42	3.22	4.83	18.33
chlorotype	a	40	97.64	155.80	24.63	47.81	147.46
	b	8	9.69	6.58	2.33	4.19	15.19
	c	40	69.31	100.52	15.89	37.16	101.45
	d	66	118.55	121.70	14.98	88.64	148.47
	np	56	24.77	62.70	8.38	7.98	41.56
stage of phenological development	flowering	39	44.14	81.33	13.02	17.77	70.50
	berry set	22	143.85	196.82	41.96	56.59	231.12
	veraison	79	72.38	102.27	11.51	49.48	95.29
	harvest	70	76.61	108.99	13.03	50.62	102.60
**Ba (µg/g)**							
area	Europe	60	21.89	16.97	2.19	17.51	26.28
	Asia	1	98.21				
	other	2	19.80	4.53	3.20	-20.86	60.46
berry color	white	16	19.52	17.78	4.44	10.05	28.99
	red	36	23.38	17.76	2.96	17.37	29.39
	np	11	27.08	25.70	7.75	9.76	44.29
chlorotype	a	15	29.13	19.61	5.06	18.27	40.00
	c	11	18.48	15.53	4.48	8.49	28.47
	d	19	18.71	15.53	3.56	11.23	26.20
	np	18	25.30	23.76	5.60	13.49	37.12
stage of phenological development	flowering	4	13.03	4.48	2.24	5.89	20.16
	berry set	7	33.51	10.61	4.01	23.71	43.32
	veraison	19	28.59	18.42	4.22	19.71	37.47
	harvest	33	18.83	20.61	3.59	11.52	26.14

				**Ni (μg/g)**			
area	Europe	57	4.79	7.88	1.04	2.70	6.88
	Asia	1	1.04				
	other	1	9.71				
berry color	white	16	8.82	13.56	3.39	1.60	16.05
	red	35	3.69	3.31	0.56	2.56	4.83
	np	20	6.10	12.59	2.81	0.21	11.99
chlorotype	a	12	3.80	3.14	0.91	1.81	5.80
	b	2	2.48	2.32	1.64	-18.36	23.32
	c	9	2.74	3.48	1.16	0.06	5.42
	d	16	5.43	3.57	0.89	3.52	7.33
	np	8	1.70	0.74	0.26	1.08	2.32
stage of phenological development	flowering	17	1.18	0.97	0.24	0.68	1.68
	berry set	5	1.98	0.43	0.19	1.44	2.52
	veraison	33	7.52	9.59	1.67	4.12	10.92
	harvest	4	1.48	0.46	0.23	0.75	2.21
				**Cr (μg/g)**			
area	Europe	35	2.47	2.57	0.43	1.59	3.35
	Asia	1	0.48				
	other	3	3.80	2.90	1.67	-3.41	11.00
berry color	white	8	3.01	2.93	1.03	0.56	5.45
	red	20	2.79	2.60	0.58	1.58	4.01
	np	11	1.67	2.26	0.68	0.16	3.19
chlorotype	a	10	3.85	2.42	0.76	2.12	5.58
	c	4	2.89	3.00	1.50	-1.88	7.66
	d	7	2.46	2.78	1.05	-0.12	5.03
	np	18	1.73	2.36	0.56	0.55	2.90
stage of phenological development	flowering	6	0.22	0.30	0.12	-0.09	0.54
	berry set	7	5.27	0.56	0.21	4.75	5.79
	veraison	22	2.64	2.64	0.56	1.47	3.81
	harvest	4	0.49	0.41	0.20	-0.16	1.14
				**Pb (μg/g)**			
area	Europe	97	3.28	6.31	0.64	2.01	4.55
	other	6	0.36	0.24	0.10	0.10	0.61
berry color	white	50	1.83	1.80	0.25	1.32	2.35
	red	37	5.11	9.63	1.58	1.90	8.32

	np	16	2.48	3.03	0.76	0.87	4.10
chlorotype	a	23	4.78	5.93	1.24	2.21	7.34
	b	1	3.56				
	c	23	1.46	1.88	0.39	0.65	2.27
	d	21	1.40	1.85	0.40	0.56	2.24
	np	35	4.12	8.95	1.51	1.04	7.19
stage of phenological development	flowering	18	3.39	2.63	0.62	2.08	4.70
	berry set	13	3.36	3.85	1.07	1.04	5.68
	veraison	24	6.09	10.72	2.19	1.56	10.62
	harvest	48	1.45	3.51	0.51	0.43	2.47
**Co (ng/g)**							
berry color	white	8	995.00	1034.38	365.71	130.24	1859.76
	red	16	1411.00	1121.34	280.34	813.48	2008.52
	np	9	391.00	637.17	212.39	-98.77	880.77
chlorotype	a	10	1816.00	1090.13	344.73	1036.17	2595.83
	c	4	875.00	876.53	438.26	-519.75	2269.75
	d	5	1122.00	917.21	410.19	-16.86	2260.86
	np	14	484.64	820.89	219.39	10.67	958.61
stage of phenological development	flowering	4	27.00	11.75	5.87	8.31	45.69
	berry set	8	2038.38	1194.48	422.31	1039.76	3036.99
	veraison	19	920.84	795.73	182.55	537.31	1304.37
	harvest	2	72.00	2.83	2.00	46.59	97.41
**Cd (ng/g)**							
berry color	white	40	308.96	639.19	101.07	104.50	513.38
	red	44	66.47	57.33	8.64	49.00	83.90
	np	10	398.49	1153.99	364.92	-427.00	1224.00
chlorotype	a	19	191.42	288.32	66.15	52.50	330.38
	c	19	108.73	128.68	29.52	46.70	170.75
	d	24	47.25	30.06	6.14	34.60	59.94
	np	32	388.47	916.56	162.03	58.00	718.93
stage of phenological development	flowering	6	78.40	85.75	35.01	-11.60	168.39
	berry set	8	194.88	96.54	34.13	114.20	275.58
	veraison	17	552.81	914.10	221.70	82.80	1022.80
	harvest	63	124.46	469.69	59.18	6.20	242.75

Table S5. Summary results of individual GLMs examining the influence of 4 categorical variables/factors (world area, stage of phenological development, berry color and chlorotype) on the concentration of individual elements. In this way, only elements that had a sufficient number of observations for group comparison (using GLMs) were processed. Effect size (Eta squared η^2) was interpreted according to Cohen (1988) as small ($\eta^2 \geq 0.01$; S), medium ($\eta^2 \geq 0.06$; M), and large ($\eta^2 \geq. 0.14$; L).

factor	degree of freedom	F statistic	p value	η^2
**K**				
intercept	1	395.98	<0.001	0.537 (L)
world area	2	15.85	<0.001	0.085 (M)
berry color	2	0.14	0.869	0.001 (-)
chlorotype	4	3.75	0.005	0.042 (S)
stage of development	3	16.19	<0.001	0.124 (M)
**Ca**				
intercept	1	221.87	<0.001	0.444 (L)
world area	2	19.23	<0.001	0.121 (M)
berry color	2	9.44	<0.001	0.064 (M)
chlorotype	4	6.25	<0.001	0.082 (M)
stage of development	3	4.19	0.006	0.043 (S)
**Mg**				
intercept	1	258.15	<0.001	0.483 (L)
world area	2	2.30	0.102	0.016 (S)
berry color	2	0.49	0.613	0.004 (-)
chlorotype	4	4.59	0.001	0.062 (M)
stage of development	3	3.96	0.009	0.041 (S)
**P**				
intercept	1	321.46	<0.001	0.554 (L)
world area	2	19.32	<0.001	0.130 (M)
berry color	2	1.29	0.278	0.010 (S)
chlorotype	4	4.41	0.002	0.064 (M)
stage of development	3	34.83	<0.001	0.287 (L)
**Na**				
intercept	1	40.11	<0.001	0.306 (L)
world area	2	20.57	<0.001	0.311 (L)
berry color	2	0.42	0.657	0.009 (-)
chlorotype	4	1.41	0.238	0.058 (S)
stage of development	3	0.80	0.498	0.026 (S)
**Fe**				
intercept	1	52.02	<0.001	0.154 (L)
world area	2	3.61	0.028	0.025 (S)
berry color	2	2.46	0.087	0.017 (S)
chlorotype	4	1.17	0.324	0.016 (S)
stage of development	3	4.40	0.005	0.044 (S)
**Al**				
intercept	1	11.72	0.001	0.190 (L)
world area	-	-	-	-

berry color	2	0.36	0.698	0.014 (S)
chlorotype	4	1.25	0.302	0.091 (M)
stage of development	3	7.78	<0.001	0.318 (L)
**Mn**				
intercept	1	94.35	<0.001	0.274 (L)
world area	2	22.38	<0.001	0.152 (L)
berry color	2	4.38	0.013	0.034 (S)
chlorotype	4	0.98	0.416	0.016 (S)
stage of development	3	4.56	0.004	0.052 (S)
**Zn**				
intercept	1	102.17	<0.001	0.263 (L)
world area	2	40.73	<0.001	0.222 (L)
berry color	2	1.63	0.198	0.011 (S)
chlorotype	4	1.27	0.280	0.018 (S)
stage of development	3	10.52	<0.001	0.099 (M)
**B**				
intercept	1	36.17	<0.001	0.187 (L)
world area	2	0.44	0.646	0.006 (-)
berry color	2	1.63	0.199	0.020 (S)
chlorotype	4	3.18	0.015	0.075 (M)
stage of development	3	4.78	0.003	0.084 (M)
**Sr**				
intercept	1	18.67	<0.001	0.293 (L)
world area	-	-	-	-
berry color	2	0.13	0.877	0.006 (-)
chlorotype	3	0.16	0.921	0.011 (S)
stage of development	2	5.64	0.007	0.200 (L)
**Cu**				
intercept	1	7.18	0.008	0.035 (S)
world area	2	5.59	0.004	0.053 (S)
berry color	2	4.73	0.010	0.046 (S)
chlorotype	4	5.17	0.001	0.095 (M)
stage of development	3	4.00	0.009	0.057 (S)
**Ba**				
intercept	1	30.56	<0.001	0.370 (L)
world area	2	13.05	<0.001	0.334 (L)
berry color	2	0.39	0.679	0.015 (S)
chlorotype	3	0.34	0.795	0.019 (S)
stage of development	3	3.77	0.016	0.179 (L)
**Ni**				
intercept	1	2.38	0.129	0.048 (S)
world area	2	0.52	0.597	0.022 (S)
berry color	2	5.82	0.006	0.199 (L)
chlorotype	4	1.38	0.257	0.105 (M)
stage of development	3	4.52	0.007	0.224 (L)
**Cr**				
intercept	1	9.04	0.006	0.244 (L)
world area	2	0.25	0.781	0.017 (S)
berry color	2	5.31	0.011	0.275 (L)
chlorotype	3	0.58	0.632	0.059 (S)
stage of development	3	8.89	0.000	0.488 (L)

		**Pb**		
intercept	1	1.56	0.215	0.017 (S)
world area	1	0.99	0.323	0.011 (S)
berry color	2	7.31	0.001	0.137 (M)
chlorotype	4	2.22	0.073	0.088 (M)
stage of development	3	3.06	0.032	0.091 (M)
		**Co**		
intercept	1	16.40	<0.001	0.406 (L)
berry color	2	0.21	0.809	0.017 (S)
chlorotype	3	0.87	0.472	0.098 (M)
stage of development	3	3.66	0.026	0.314 (L)
		**Cd**		
intercept	1	19.30	<0.001	0.189 (L)
berry color	2	5.15	0.008	0.110 (M)
chlorotype	3	1.25	0.297	0.043 (S)
stage of development	3	5.10	0.003	0.156 (L)

Table S6. The ratio of individual elements in leaves to those in soil (so-called bioaccumulation factor, mean value) calculated based on the quantitative data shown in Table 2 of the main text. Green color indicates elements with values close to or greater than 1, and blue color indicates elements with values in the range of approximately 0.1 – 0.6. leaves of white grapevine (Rhein Riesling) and red grapevine (Cabernet Sauvignon) were collected in two ontogenetic stages (young and mature leaves as shown on the next page). Values within a row sharing the same letter(s) are not significantly different according to one-way ANOVA followed by Tukey's post-hoc test ($p > 0.05$).

	**white leaves**		**red leaves**	
	young	mature	young	mature
K	1.73a	0.85b	1.53a	0.93b
Ca	0.13b	0.15b	0.072c	0.18a
P	6.61a	3.15b	5.27a	3.34b
Mg	0.22a	0.17b	0.12c	0.17b
Fe	0.0022c	0.0035a	0.0023c	0.0030b
Zn	0.66a	0.34b	0.60a	0.31b
B	1.10a	1.05a	0.76b	1.08a
Mn	0.060c	0.086bc	0.096b	0.15a
Rb	0.42a	0.14c	0.32b	0.039d
Na	0.0031a	0.0019b	0.0033a	0.0017b
Al	0.00041c	0.0013a	0.00022d	0.00066b
Cu	0.49a	0.38b	0.41b	0.38b
Sr	0.068c	0.088b	0.042d	0.12a
Ba	0.0084c	0.010b	0.0080c	0.014a
Ni	0.087a	0.083a	0.053b	0.028b
Ti	0.00035b	0.00092a	0.00022b	0.0010a
Cr	0.010b	0.021a	0.0095b	0.011b
Mo	0.38a	0.50a	0.15b	0.15b
Cs	0.026a	0.014b	0.011c	0.0033d
Sb	0.042c	0.15a	0.029c	0.12b
V	0.00057c	0.0017a	0.00034d	0.00098b
Pb	0.0022b	0.0070a	0.0014b	0.0066a
Se	0.0051c	0.0065bc	0.0079b	0.013a
Li	0.00098b	0.0024a	0.00049c	0.0011b
Co	0.0031b	0.0046a	0.0025b	0.0026b
Sn	0.0049b	0.0083a	0.0037b	0.0072a
As	0.00030c	0.00057a	0.00026c	0.00039b
Zr	0.00043b	0.0011a	0.00032b	0.00084a
Cd	0.0083bc	0.013a	0.0057c	0.0099b
Be	0.00059bc	0.0012a	0.00036c	0.00080b

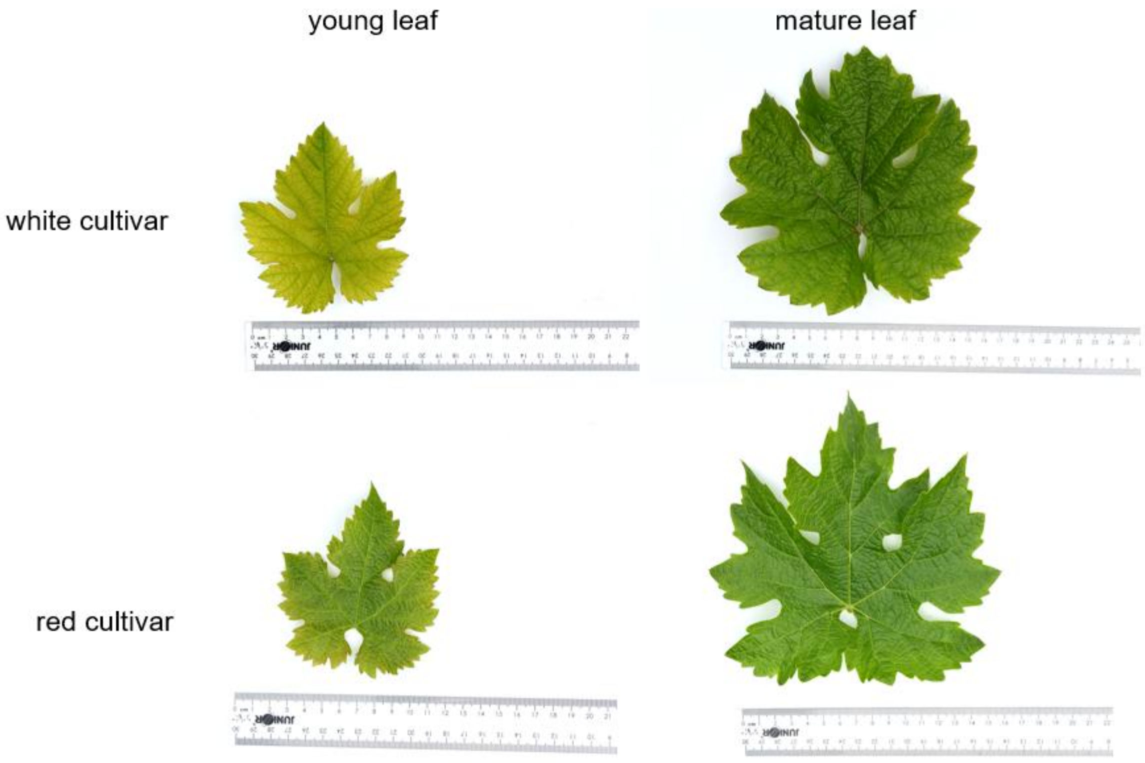

Figure S8. Representative photos of young and mature leaves of our authentic cultivars harvested in May 2025 (white cultivar Rhein Riesling and red cultivar Cabernet Sauvignon). The difference in leaf area between young and mature leaves is about 3-fold and the difference in weight is about 4-fold for both cultivars.

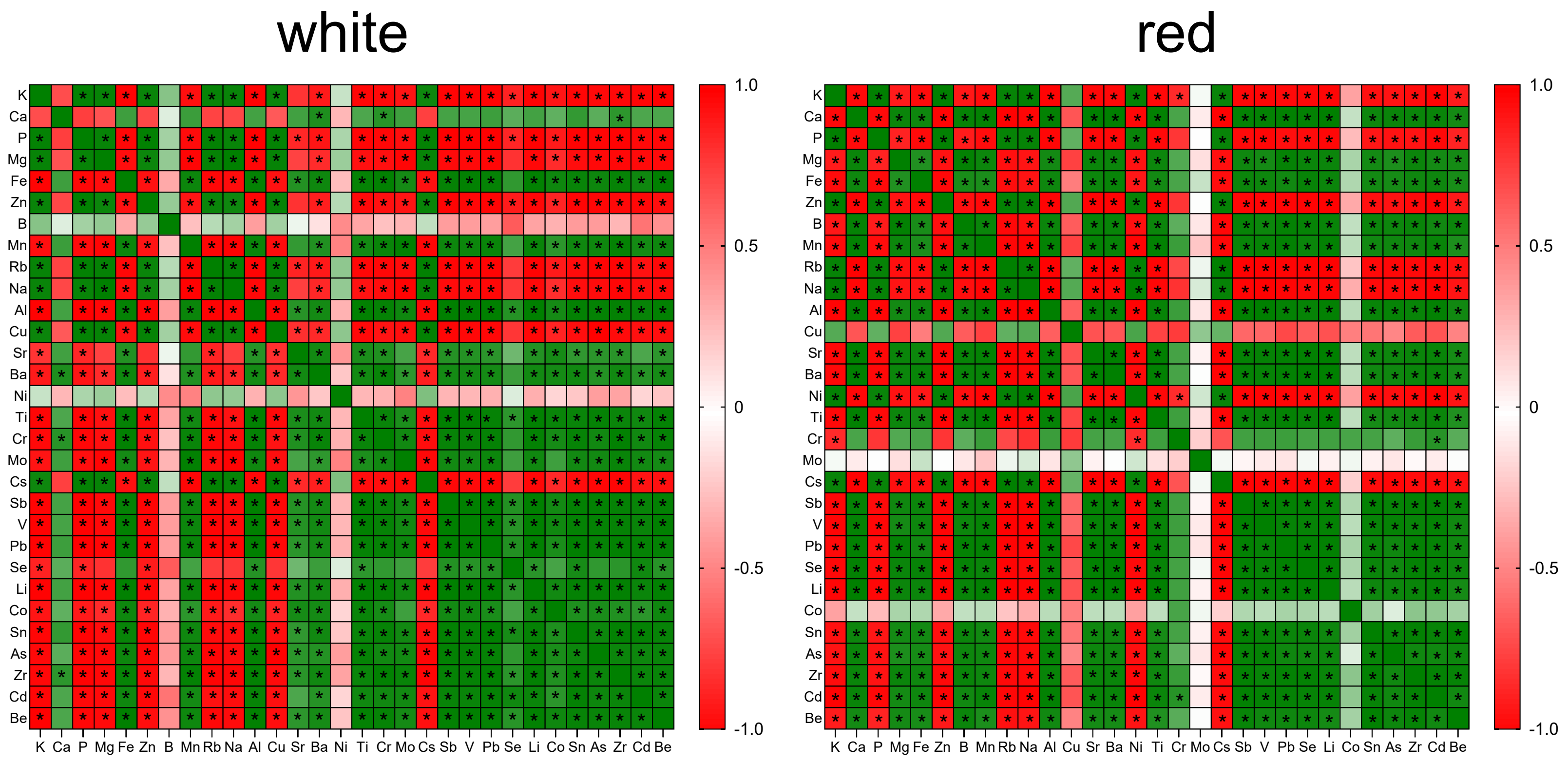

Figure S9. Pearson's correlation analyses between the concentration of elements in our authentic leaf samples: white cultivar (Rhein Riesling) and red cultivar (Cabernet Sauvignon). Correlations were considered significant at $p < 0.05$. The green and red squares indicate positive and negative correlation, respectively. We note that the correlation of Mg with many elements in particular differs in a mirror-like manner between cultivars.

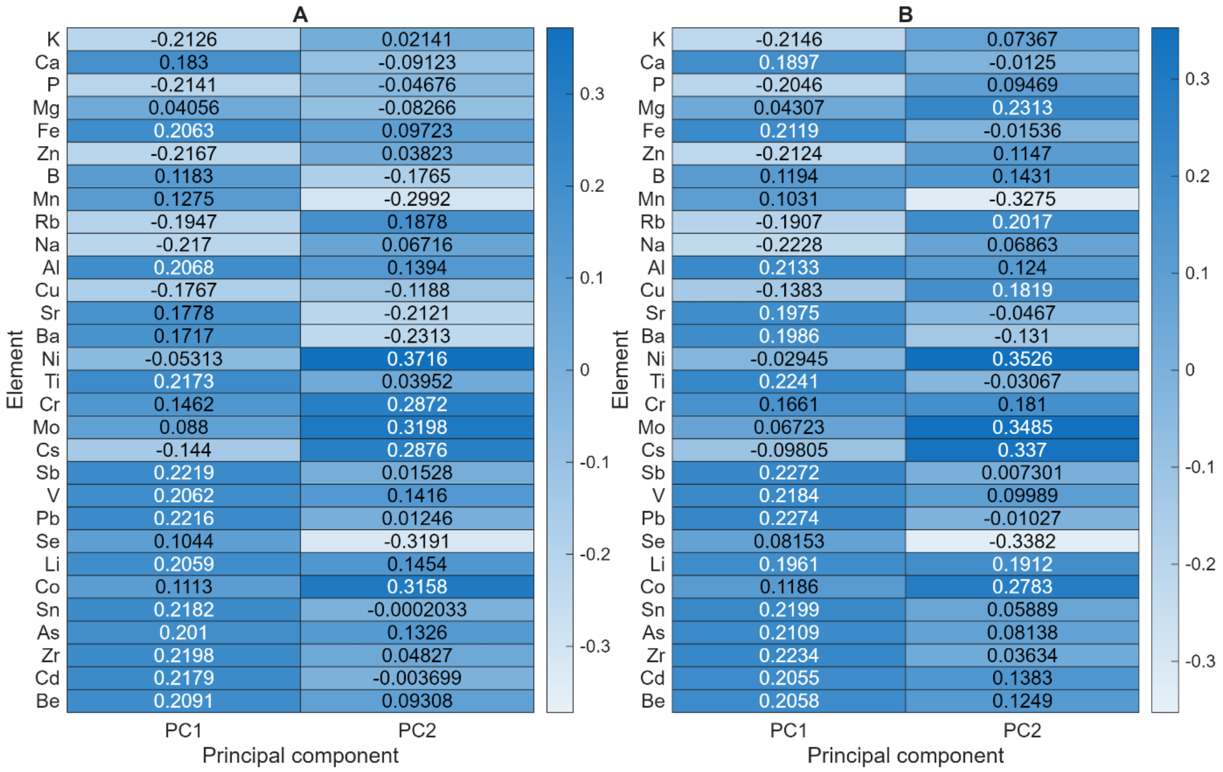

Figure S10. Heatmaps of loading values for the first two principal components obtained from principal component analysis (PCA) of grapevine leaves: (A) elemental concentrations and (B) bioaccumulation factors (BAF). The heatmaps illustrate the relative contribution of individual elements to PC1 and PC2, and the corresponding PCA plot is shown in Figure 3 of the main text.
